# GNAT family Pat2 is required for long-term survival on glycerol and catalyzes lysine acetylation of glycerol kinase in hypersaline-adapted archaea

**DOI:** 10.1128/mbio.02514-25

**Published:** 2025-10-27

**Authors:** Heather N. Judd, Karol M. Sanchez, Leah S. Dublino, Gabriel J. Zhang, David Yu, Daniel Gal, Ricardo L. Couto-Rodríguez, Xin Wang, Julie A. Maupin-Furlow

**Affiliations:** 1 Department of Microbiology and Cell Science, Institute of Food and Agricultural Sciences, University of Florida 3463, Gainesville, Florida, USA; 2 Genetics Institute, University of Florida 3463, Gainesville, Florida, USA; Albert-Ludwigs-Universitat Freiburg, Freiburg, Germany

**Keywords:** archaea, halophiles, post-translational modification, lysine acetylation, glycerol metabolism, glycerol kinase, regulation

## Abstract

Lysine acetylation is a widespread post-translational modification that regulates key biological processes including metabolism and chromatin dynamics, yet its roles in archaea remain poorly understood. Here, we investigated two Gcn5-related N-acetyltransferase (GNAT) family homologs, *pat1* and *pat2*, in the halophilic archaeon *Haloferax volcanii* (Hv). A ∆*pat2* mutant exhibited premature cell death on glycerol, a phenotype not observed in the parent strain, ∆*pat1* mutant, or on glucose. Complementation of the ∆*pat2* mutant with plasmid-expressed *pat2* restored survival on glycerol, confirming the essential role of *pat2* in this process. *In vitro* assays revealed HvPat2 catalyzes lysine acetylation of glycerol kinase, HvGlpK, an enzyme required for growth on glycerol. Computational modeling predicted that HvPat2 residues E105, Y154, V110, and N147 may form hydrogen bonds with acetyl-CoA. To assess the importance of these residues, alanine substitutions were introduced. N147A and V110A complemented the ∆*pat2* mutant for survival on glycerol but showed little or no activity in acetylating HvGlpK *in vitro*. E105A and Y154A had the most pronounced effects, failing to restore ∆*pat2* survival on glycerol and showing no catalytic activity toward HvGlpK acetylation. Moreover, the Y154A variant co-purified with HVO_2384, a tandem CBS domain protein with a C-terminal ribosome hibernation factor domain, suggesting an additional regulatory interaction. These findings highlight the critical role of the GNAT HvPat2 in survival on glycerol, reveal its ability to acetylate a central glycerol metabolism enzyme in archaea, and offer mechanistic insight into GNAT family acetyltransferases.

**IMPORTANCE:**

GNAT family homologs are widespread and diverse in their use of acyl-CoAs to acylate small molecules and proteins, functions difficult to predict based on *in silico* analysis alone. Here, we reveal a critical role for lysine acetylation in archaeal central carbon metabolism, identifying the GNAT family Pat2 of *Haloferax volcanii* as essential for long-term survival on glycerol (compared to glucose) and capable of mediating the lysine acetylation of glycerol kinase, a key enzyme in glycerol metabolism. Pat2 residues important for catalytic activity and a putative regulatory partner (HVO_2384) are also identified. The findings expand our understanding of GNAT family acyltransferases and highlight conserved mechanisms of metabolic control by post-translational modification across domains of life.

## INTRODUCTION

Lysine acetylation (Kac) is an ancient form of post-translational modification (PTM) common to all domains of life that regulates metabolism, chromatin remodeling, transcription, cell structure, and other important biological functions (1, 2). Fundamental to Kac is the addition of an acetyl group to the epsilon amino (*N*^ɛ^) group of lysine residues which neutralizes a positive charge. This covalent modification can impact DNA binding, protein-protein interactions, enzyme activity, substrate binding, and protein stability (3, 4). Kac occurs spontaneously through pools of reactive acetyl-CoA and acetyl-P or enzymatically through lysine acetyl transferases (KATs). Acetyl groups are removed from lysine residues by NAD^+^-dependent sirtuins (SIRTs; silent information regulators) and Zn^2+^-dependent lysine deacetylases (KDACs or HDACs) (5, 6).

KATs cluster to the GCN5-related N-acetyltransferase (GNAT) superfamily (7–9). GNATs are diverse and widespread (10) with representative members including not only KATs but also enzymes that catalyze the N-terminal (N^α^-) acetylation of proteins (11), lysine succinylation (12), the covalent modification of non-protein substrates such as aminoglycoside antibiotics (1–3), and other activities. One of the first KATs identified was the *Salmonella enterica* Pat shown to acetylate lysine residues of acetyl-CoA synthase (ACS), a central enzyme of acetate metabolism (4). Similar KAT-ACS connections are observed in *Escherichia coli*, *Bacillus subtilis*, *Rhodopseudomonas palustris*, *Streptomyces coelicolor*, *Mycobacterium smegmatis*, and *M. tuberculosis* (5–9). Many of the bacterial KAT representatives have a regulatory domain(s) fused to the GNAT catalytic domain (13). The *E. coli* PatZ and *Salmonella enterica* SePat are examples of KATs fused to a nucleoside diphosphate (NDP)-forming CoA ligase-related domain (6). Likewise, *M. tuberculosis* bKAT has an allosteric cyclic AMP (cAMP)-binding domain (14), and *Micromonospora aurantiaca* bKAT features an amino acid-sensing domain (15). In yeast, many GNATs exist with regulatory domains or as multisubunit complexes (16, 17). By comparison, archaeal GNATs are typically single-domain proteins (Pfam PF00583).

While diverse, GNATs have a common catalytic core. This core includes a classical secondary structure consisting of six to seven β-strands and four to five α-helices where a β-bulge is formed around the β4-strand and the acetyl-CoA-binding site is formed by a V-like cleft between the β4 and β5 strands (18). Acetyl-CoA binding is facilitated by the phosphate binding loop positioned around β4 and α3, called the P-loop, which is formed by a conserved set of residues (Q/R-x-x-G-x-G/A) where x is any amino acid, and the nitrogenous backbone stabilizes the pyrophosphate moiety of coenzyme A (18).

Given their distinct phylogenetic relationship to bacteria and eukaryotes, archaea serve as excellent models for studying the evolution of protein function; however, biochemical studies on archaeal members of the GNAT superfamily remain limited. Although a 2Fe-2S ferredoxin of *Halobacterium salinarum* is one of the first reports of a lysine acetylated protein in prokaryotes (19), the KAT catalyzing this Kac remains to be determined. Instead, much of the current knowledge of archaeal GNATs and lysine acetylation is from the work of *Sulfolobus solfataricus* SsPat that acetylates Alba at K16, decreasing the nucleic acid binding affinity of this chromatin remodeling protein (20, 21). In *Haloferax volcanii,* HvPat1 (HVO_1756), HvPat2 (HVO_1821), and Elp3 (HVO_2888) are identified as GNAT homologs (22). These *H. volcanii* GNATs have limited primary amino acid sequence homology to the SsPat. In a proteomics study, the abundance of HvPat1 and HvPat2 is found inversely correlated with HvPat1 being downregulated during hypochlorite stress, whereas HvPat2 is upregulated (23).

In this study, we investigated the function of the GNAT family lysine acetyltransferases HvPat1 and HvPat2 in the model archaeon *Haloferax volcanii*. We focus on understanding the role of these enzymes in central carbon metabolism, particularly during growth on glycerol. Through genetic, biochemical, and structural analyses, we (i) identify Pat2 as essential for long-term survival on glycerol compared to glucose; (ii) demonstrate that Pat2 catalyzes lysine acetylation of glycerol kinase (HvGlpK), a central enzyme required for growth on glycerol; (iii) define key residues within Pat2 required for its catalytic activity, expanding our understanding of GNAT domain structure and function; and (iv) find that an inactive Pat2 variant co-purifies with HVO_2384, a CBS domain protein with a ribosome hibernation factor domain, suggesting additional layers of regulation. Our findings provide new insights into the functional significance of lysine acetylation in archaea and broaden our understanding of GNAT family enzymes in metabolic regulation.

## MATERIALS AND METHODS

### Materials

Biochemicals were purchased from Fisher Scientific (Atlanta, GA, USA), Bio-Rad (Hercules, CA, USA), and Sigma-Aldrich (St. Louis, MO, USA). Oligonucleotides and DNA Sanger sequencing services were purchased from Eurofins Genomics (Louisville, KY, USA). Phusion High-Fidelity DNA Polymerase and other DNA modification enzymes were purchased from New England Biolabs (NEB) (Ipswich, MA, USA). DNA fragments were isolated using NEB Monarch PCR & DNA Cleanup Kit and DNA Gel Extraction Kit (Ipswich, MA, USA). Acetyl-CoA (cat. No. 16160) was purchased from Cayman Chemical (Ann Arbor, Michigan, USA).

### Strains, media, and growth conditions

Strains used in this study are summarized in Table S1. *E. coli* cultures were grown at 37°C in Luria-Bertani (LB) medium supplemented with ampicillin (100 µg/mL) (Amp) and/or chloramphenicol (34 µg/mL) (Cam) as needed. *H. volcanii* strains were grown at 42°C in ATCC974, Hv-CA+ or minimal media as previously described with the following modifications (24). Minimal medium was composed of 18% salt water, 30 mM Tris-HCl, pH 7.5, 3 mM CaCl_2_, 10 mM NH_4_Cl, 3 mM of desired carbon source (glycerol, fructose, or glucose), 0.975 mM potassium phosphate buffer, pH 7.5, and 50 µg/mL uracil along with trace elements, thiamine, and biotin at concentrations as previously described (24). *H. volcanii* medium was supplemented with novobiocin (0.2 µg/mL), uracil (50 µg/mL), and/or tryptophan (2 mM) as needed. Liquid cultures were grown by angled rotation in culture tubes (13 × 100 mm^2^) (Glas-Col mini-rotator, Terre Haute, IN) or continuous orbital shaking at 200 rpm (New Brunswick Scientific I 24 Incubator Shaker, Edison, NJ) as indicated. Culture plates were supplemented with 1.5% (wt/vol) agar. Growth was measured by optical density at 600 nm (OD_600_) where 1 OD_600_ unit equals approximately 10^9^ CFU/mL. All strains, including those carrying plasmids, were stored in 20% (vol/vol) glycerol stocks at −80°C until use.

### Strain and plasmid constructions

Plasmids and primers synthesized and used in this study are summarized in Table S1. For all plasmid constructions, DNA fragments for cloning were generated by PCR using high-fidelity Phusion DNA polymerase. The genomic DNA used for PCR template was extracted from *H. volcanii* H26 following the protocol outlined in *The Halohandbook* (24). PCR products were purified using the Monarch PCR and DNA Cleanup Kit (New England Biolabs). PCR products were digested with restriction enzymes (as indicated in Table S1) according to the manufacturer’s protocols. After digestion, the DNA fragments were cleaned using PCR clean-up (New England Biolabs) and ligated using Quick T4 DNA ligase at room temperature (RT) for 15 min. DNA ligation products were transformed into *E. coli* Top10. Plasmid DNA was extracted into DNase/RNase-free deionized water (ThermoFisher) using the PureLink Miniprep Kit (Invitrogen). PCR was used for screening, and Sanger DNA sequencing was used to verify the fidelity of the plasmid insert (Eurofins Genomics, Louisville, KY). Plasmids were transformed into the *dam^−^ dcm^−^* strain *E. coli* GM2163 to remove methylation of the DNA prior to transformation of *H. volcanii* strains according to the *Halohandbook* (24). Selection markers for *H. volcanii* included *gyrB* (encoding a modified GyrB subunit of DNA gyrase rendering cells resistant to novobiocin) and *pyrE2* (encoding orotate phosphoribosyl transferase rendering *ΔpyrE2* strains viable in Hv-CA+ medium devoid of uracil).

To generate the *pat2-SII* expression plasmids (encoding HvPat2 fused to a C-terminal StrepII tag), the *pat2* gene was PCR amplified from *H. volcanii* genomic DNA using primers HVO_1821_NdeI and HVO_1821-KpnI. The PCR product was ligated into the NdeI and KpnI sites of pJAM809 to generate plasmid pJAM4017 carrying the *pat2-SII* gene under control of the P2*_rrnA_* promoter. The *pat2-SII* gene was amplified from pJAM4017 by PCR using Pat2_ecori_F and Pat1_rev primers. The PCR product was ligated into the NdeI and BamHI sites of pTA963 to generate plasmid pJAM4554, carrying the *pat2-SII* gene under control of the tryptophan-inducible promoter P*_tnaA_*.

Site-directed mutations (SDMs) of *pat2-SII* were generated on pJAM4017 using pat2_SDM-anchor1, pat2_SDM-anchor2, E105A_rev, V110A_rev, N147A_rev, and Y154A_rev primers using the rrPCR method as previously described (25). The resulting plasmids included pJAM4555 (Pat2-SII E105A), pJAM4556 (Pat2-SII V110A), pJAM4557 (Pat2-SII N147A), and pJAM4558 (Pat2-SII Y154A). All *pat2-SII* wild-type and SDM plasmids were transformed into *H. volcanii* strain JM502 (H26 ∆*pat2*).

The *glpK* gene was isolated from *H. volcanii* genomic DNA by PCR using primers F-glpK and R-glpK. The 1.5 kb PCR fragment was digested, purified, and ligated into the NdeI and BlpI sites of plasmid pET15b. The resulting plasmid, pJAM4360 (pET15b expressing *Hv*GlpK), was transformed in *E. coli* Top10 for storage in the 20% (vol/vol) glycerol stocks at −80°C.

### Growth curves and spot plates

Strains were streaked from the −80°C stored 20% (vol/vol) glycerol stocks onto ATCC974-rich medium plates and incubated for ~7 days at 42°C. Novobiocin was included in the medium for strains carrying the empty vector (pJAM202C) and *pat2-SII* expression plasmids (pJAM4017, pJAM4555, pJAM4556, pJAM4557, and pJAM4558). A single isolated colony was used to inoculate 5 mL of minimal medium supplemented with the designated carbon source (glucose or glycerol) and cultured for 2 days in rotating culture tubes at 42°C. Cells were then diluted to an optical density (OD_600_) of 0.02 in 5 mL of fresh medium (glucose or glycerol minimal medium) and incubated again at 42°C overnight in rotating culture tubes. The following morning, cells were diluted to an OD_600_ of 0.02 again in 5 mL fresh medium (glucose or glycerol minimal medium) and then rotated in the culture tubes at 42°C for 15 min for mixing. Cells were added to a sterile polystyrene tissue cultured treated 96-well, flat-bottom cell culture plate (reproducible results with either CellPro, Alkali Scientific, Fort Lauderdale, FL, or GenClone, Genesee Scientific, El Cajon, CA), where each well had a final volume of 200 µL (5 replicates per strain per media type; glucose or glycerol minimal medium) with outer wells containing 300 µL of sterile diH_2_O. Wells with each medium type were also included as a blank control to account for background signal. The lid was secured to the plate bottom using 3M micropore tape. Growth curves were based on monitoring OD_600_ every 15 min for 120 h at 42°C with aeration (continuous orbital shaking) using a microtiter plate reader (BioTek Epoch 2, Agilent Technologies, Santa Clara, CA). After the 120 h incubation on the minimal medium (glucose or glycerol minimal medium), the strains were serially diluted (10^−1^, 10^−2^, 10^−3^, and 10^−4^) and spotted (20 µL) onto agar plates. The type of media in the agar plates used for the serial dilution was varied and included glucose minimal, glycerol minimal, or ATCC-rich medium.

### Large-scale cultivation of *H. volcanii* strains for Pat2 purification

Pat2 and variant proteins were purified from *H. volcanii* H26 ∆*pat2* strains carrying the *pat2-SII* and SDM expression plasmids. For strains carrying plasmids with the *pyrE2*^+^ marker (e.g*.,* pJAM4554), cells were freshly streaked from glycerol stocks onto Hv-CA+ agar plates. A single colony was used to inoculate 100 mL Hv-CA+ medium and incubated for ~2 days at 42°C with orbital shaking in 500 mL Erlenmeyer flasks. The starter culture was subcultured (10 mL) into 500 mL of Hv-CA+ and incubated at 42°C with orbital shaking in 2.8 L Fernbach flasks until OD_600_ reached 0.6, where expression of *pat2-SII* ORF was induced by the addition of 2 mM L-tryptophan and cultured for an additional 12–18 h. Once the desired OD_600_ of 1.2–1.4 was reached, cells were harvested by centrifugation (2,995 × *g* at 20°C for 50 min). Cell pellets were stored at −80°C until use. For strains carrying plasmids with the *gyrB* (Nov^R^) marker (e.g.*,* pJAM4017 and derivatives), the cells were cultured in a similar manner except ATCC974 supplemented with 0.3 µg/mL novobiocin was used for selection instead of Hv-CA+, and no L-tryptophan was needed for induction of the *pat2-SII* and SDMs.

### Pat2 purification

Cell pellet (usually 3 g wet weight) was resuspended in 15 mL lysis buffer (50 mM HEPES, pH 7.5, 2 M NaCl, 1 mM TCEP, 3 mM MgCl_2_, 1 mM CaCl_2_, 10 µg/mL DNase I, and Mini cOmplete EDTA-free protease inhibitor tablets (used according to manufacturer’s recommendations). Cells were disrupted using a French pressure cell with three to four passes (1,500 psi, minimum high ratio of 140, Glen-Mills, NJ, USA). Lysate was clarified by centrifugation (13,177 *× g* at 4°C for 30 min) and filtration (0.45 µm and 0.22 µm, PES filters, CellPro). Samples were incubated with 0.5 mL (1 mL 50% slurry) Strep-Tactin Superflow Plus resin (Qiagen, Germantown, MD) in 10 mL columns (Pierce, ThermoFisher Scientific, USA, Cat. No. 89898) for at least 1 h at 4°C with gentle rocking. Prior to sample application, resin was equilibrated in wash buffer (50 mM HEPES, pH 7.5, 2 M NaCl, 1 mM TCEP). After the application of the sample, non-specific proteins were removed by subsequent rounds of washing with the wash buffer (5 washes with 2.5 mL wash buffer). Pat2-SII and variant proteins were eluted from the column by incubating with 300 µL wash buffer supplemented with 1 mM desthiobiotin for 30 min at 4°C, two times total for a final volume of 600 µL. Final elutions (600 µL total) were dialyzed at 4°C overnight in dialysis buffer (50 mM HEPES, pH 7.5, 2 M NaCl, 1 mM DL-1,4-dithiothreitol [Thermo Fisher, USA]) using D-Tube dialyzer midi, MWCO 3.5 kDa (EMD Millipore Corporation, Burlington, MA, USA). D-tubes were prepared according to the manufacturer’s directions prior to use. Pat2 fractions were combined and concentrated to ≤500 µL using Sartorius Vivaspin Turbo 5 10,000 MWCO centrifugal concentrator columns according to the manufacturer’s directions (Fisher Scientific, USA) and then further purified by size-exclusion chromatography (SEC) using a Superdex 75 Increase 10/300 Gl FPLC column (Cytiva, Marlborough, MA). Aliquots of protein samples (0.5 mL) were applied to the column equilibrated in freshly prepared filtered dialysis buffer (see above) at a flow rate of 0.3 mL/min. Fractions (0.5 mL) were collected and analyzed for Pat2 protein. The final fractions were combined and concentrated using a Vivaspin centrifugal concentrator (3 MWCO, MilliporeSigma, Burlington, MA).

### Protein quantification and SDS-PAGE

Protein concentration was measured by Bradford assay with bovine serum albumin as the standard according to the manufacturer’s protocol (Bio-Rad, Hercules, CA). For each measurement, a sample (5 µL) was mixed with 250 µL of Bradford reagent, and the mixture was incubated for 5 min at RT. Absorbance at 595 nm (A595) was recorded using a BioTek Epoch 2 microtiter plate reader. The assay exhibited linearity within the 0 to 2,000 µg∙mL⁻¹ protein range. For SDS-PAGE analysis, proteins were mixed with 2× SDS-PAGE loading buffer: 100 mM Tris-HCl buffer, pH 6.8, 4% (wt/vol) SDS, 20% (vol/vol) glycerol, 0.6 mg/mL bromophenol blue, and 5% (vol/vol) β-mercaptoethanol. Samples were boiled for 5–10 min and separated by reducing 12% SDS-PAGE in Tris-glycine-SDS (TGS) buffer. Precision Plus Protein Kaleidoscope molecular mass marker (BioRad, Hercules, CA) was used as the standard. Proteins were stained in gel with Coomassie Brilliant Blue R-250 for 1 h at RT and then destained with diH_2_O overnight. Gels were imaged using an iBright Imaging System (Invitrogen, Carlsbad, CA) according to the manufacturer’s protocol.

### Immunoblotting analysis

Proteins were transferred from SDS-PAGE gels to PVDF membranes (0.45 µm) (Immobilon-FL, Millipore, Burlington, MA) using the wet transfer method at 30 V for 14 h at 4°C with constant stirring as per standard protocol (BioRad, Hercules, CA, USA). After protein transfer, the membranes were incubated with gentle rocking for 2 h at RT in blocking buffer composed of TBST (0.05 M Tris-HCl, pH 7.6, 0.15 M NaCl, 0.1% [vol/vol] Tween 20) supplemented with 5% (wt/vol) skim milk powder for immunoblotting analysis of His-tagged and SII-tagged proteins. To detect His-tagged proteins, the membranes were incubated at RT for 1 h with a 1:10,000 dilution of HRP-conjugated mouse monoclonal anti-6× His tag antibody (Cat. No. HRP-660005). After incubation, the membranes were washed 5 × 5 min with TBST. To detect SII-tagged proteins, membranes were incubated at RT for 1 h with polyclonal anti-SII tag antibody (NWSHPQFEK Genescript A002026 rabbit) at 0.25 µg per mL blocking buffer. After washing the membrane 5 × 5 min with TBST, the membrane was incubated for 1 h at RT with a 1:10,000 dilution of goat anti-rabbit HRP-conjugated (Southern Biotech #4010-05). The membrane was similarly washed 5 × 5 min with TBST. To detect lysine acetylation, membranes were blocked overnight at 4°C in TBST supplemented with 5% (wt/vol) BSA and probed with anti-acetyllysine rabbit mAb (PTM Bio, #PTM-105RM) as the primary at 1:5,000 dilution and mouse anti-rabbit IgG-HRP (Santa Cruz Biotechnology; #sc-2357) as the secondary antibody at 1:10,000 dilution. Chemiluminescent signals were detected using Chemiluminescent solution according to the supplier (Thermo Scientific, #89880) on an iBright FL1000 Imaging System (Thermo Fisher Scientific).

### HvGlpK purification from recombinant *E. coli*

*E. coli* Rosetta (DE3) was freshly transformed with the His-HvGlpK expression plasmid pJAM4360 and selected on LB Amp/Cam plates. Isolated colonies were used to inoculate a starter culture in 20 mL LB Amp/Cam (250 mL Erlenmeyer flask). The starter culture was grown at 37°C until an OD_600_ of 0.8 was reached. Subsequently, 500 mL of large-scale cultures was inoculated to an initial OD_600_ of 0.01 and incubated at 37°C with orbital shaking. When the cultures reached an OD_600_ of 0.6–0.8, the temperature was reduced to 25°C. Once the cultures reached 25°C, protein expression was induced by adding isopropyl-β-D-1-thiogalactopyranoside (IPTG) to a final concentration of 0.4 mM, followed by overnight incubation with orbital shaking. After 16 h, cells were harvested, and pellets were either stored at −80°C or immediately resuspended in lysis buffer (50 mM HEPES, pH 7.5, 50 mM NaCl, 5 mM β-mercaptoethanol, 40 mM imidazole, 10 µg/ mL DNase, and protease inhibitors) at a ratio of 5 mL per gram of cells. IPTG was added only after the cells reached 25°C to maintain protein solubility, and pellets were resuspended and lysed in low-salt buffer before buffer exchange to high-salt conditions (2 M NaCl) using Zeba spin desalting columns 7K MWCO according to the manufacturer’s buffer exchange protocol (Thermo Scientific, Rockford, IL). This step was introduced to minimize aggregation of the “salt-loving” GlpK. Ni(II)-NTA His-Bind resin (0.25 mL of 50% slurry; EMD Millipore Corporation, Burlington, MA, USA) was equilibrated in wash buffer composed of 50 mM HEPES, pH 7.5, 2 M NaCl, 5 mM β-mercaptoethanol, and 40 mM imidazole. Cell lysate (0.5 mL) was incubated with the pre-equilibrated resin for 1 h at 4°C with gentle rocking. The resin with the sample was washed three times with wash buffer (50 mM HEPES, pH 7.5, 2 M NaCl, 40 mM imidazole) and eluted two times with 200 µL elution buffer (50 mM HEPES, pH 7.5, 2 M NaCl, 250 mM imidazole) for a final elution volume of 400 µL. Dialysis was performed as previously described for Strep-II tag purifications, except that 10% (wt/vol) glycerol was included in the HvGlpK dialysis buffer.

### Lysine acetylation activity assay

Lysine acetylation reactions (50 µL) contained the following: 6 µM enzyme (HvPat2), 2 µM substrate protein (HvGlpK_Ec_), 0.2 mM acetyl-CoA, 50 mM HEPES, pH 7.5, 2 M NaCl, and 1 mM DTT. Reactions were incubated at 37°C for 3 h followed by precipitation overnight on ice with 10% (wt/vol) trichloroacetic acid (TCA). Samples were centrifuged (10 min, 17,999 × *g*, 4°C), and protein pellets were washed with 100% cold acetone at 4°C. Air-dried protein pellets were resuspended in 20 µL 2× SDS-PAGE reducing buffer, boiled for 5 min, and resolved by 12% SDS-PAGE for Coomassie blue staining and immunoblotting as previously described.

### *In silico* modeling of HvPat2 and docking of acetyl-CoA and HvGlpK

Residues involved in binding acetyl-CoA were identified by first finding GNATs with structural homology to HvPat2 (PDB and GeneBank accession numbers: 4NXY, AAG08251.1, AEW61585.1, ANO32847.1, 2I79). These GNATs were identified using AlphaFold Foldseek (https://search.foldseek.com/search). Clustal Omega (26) was used to align the primary sequences. The resulting .aln file and the predicted Pat2 structure were imported to ESPript 3.0 webserver (https://espript.ibcp.fr/ESPript/ESPript/) (27) to align the crystal and predicted structures using HvPat2 as a reference to predict conserved secondary structure formation among the data set. The predicted tertiary structure of HvPat2 was superimposed onto the crystal PDB structure of *S. lividans* PatA (PDB: 4NXY) bound to acetyl-CoA using the ChimeraX matchmaker structural tool (https://www.cgl.ucsf.edu/chimerax/). This overlay was used to identify residues for acetyl-CoA binding, which was later used in the docking analysis. To model molecular docking, the PDB file of the HvPat2 model was modified using ChimeraX Dock Prep. This step is necessary to ensure the enzyme is in the proper format for optimized molecular docking simulations by removing water molecules and non-protein ligands, adding necessary hydrogens, and assigning partial charges. Once prepared, the HvPat2 PDB file was uploaded to PyRx (https://pyrx.sourceforge.io/) alongside the crystal structure of acetyl-CoA (PubChem CID: 444493). Docking was performed on the involved residues, followed by energy minimization of the overall docked structure to produce the most energetically efficient binding conformation. Upon completion, the program returned models of docked acetyl-CoA: HvPat2 poses ranked based on binding energy (kcal/mol). The most energetically efficient model was chosen and visualized in ChimeraX. AlphaFold 3 was used to predict HvPat2:HvGlpK binding.

### Statistical analysis of growth curves and enzymatic activity

Growth curve, long-term survival spot plating, and enzymatic activity assays were performed under defined conditions (as outlined in earlier sections). Each growth curve experiment included biological replicates performed in at least triplicate and was conducted a minimum of three times to ensure reproducibility. Area under the curve (AUC) values were calculated based on the trapezoidal rule and are presented as mean values ± standard deviation (SD). Statistical significance between experimental groups was assessed using Student’s *t*-test, with a threshold for significance set at *P*-value < 0.05. All statistical analyses were performed using Microsoft Excel. Enzymatic activity assay experiments were performed at least in duplicate and demonstrated to be reproducible, with representative immunoblots presented.

### In-gel digestion for LC-MS/MS analysis

Protein samples were resolved on SDS-PAGE gels at 160 V until the tracking dye migrated the desired distance. To visualize proteins, gels were rinsed in nanopure-diH_2_O for 5 min with rocking, then fixed for 30 min in a 20%–35% methanol and 10%–20% acetic acid solution (Coomassie Brilliant Blue R-250 Destaining Solution; Bio-Rad, cat. no. 1610438). Glassware used for these steps was washed with 50% methanol to reduce keratin contamination. The gel was then stained in 50 mL Bio-Safe Coomassie G-250 Stain (Bio-Rad, cat. no. 1610786) for 1 h with rocking and rinsed in nanopure-diH_2_O for at least 1 hour or overnight. For each gel slice, a 1.5 mL microfuge tube was prepared by washing two times with 50% acetonitrile (ACN, Sigma-Aldrich, cat. no. 900667, USA)/ 1% trifluoroacetic acid (TFA, Fisher Scientific, cat. no. BP618-500). The protein band co-purifying with HvPat2-SII Y154A (compared to the empty vector control) was excised from the gel using a fresh blade and clean gloves. Each gel slice was destained twice with 0.2 mL 100 mM ammonium bicarbonate (NH_4_HCO_3_) (Fisher BioReagents, Fair Lawn, NJ, USA, cat. no. BP2413-500)/50% ACN for 45 min each at 37°C, discarding the supernatant after each incubation. Gel slices were dehydrated at RT in 100 µL 100% ACN for 5 min or until they turned opaque in appearance. Gel slices were dried at RT for 15 min on a SpeedVac (Vacufuge Plus, Eppendorf). Gel slices were then rehydrated by incubating in 10 µL of trypsin (Promega, cat. no. V5280) digest solution (20 µg·mL^−1^ in 40 mM NH_4_HCO_3_/10% ACN) for 1 hour at RT until they no longer appeared opaque. Digestion buffer was added to the tube to just cover the gel slice. Microtubes were sealed tightly to prevent evaporation and then incubated at 37°C overnight. In the same tube with the digestion buffer, the gel slices were rinsed twice with nanopure-diH_2_O by incubating for 10 min with mixing via vortex every 2 min. The supernatant was transferred to a new tube. Digested peptides in the gel slice were extracted by mixing with 50 µL 50% ACN/ 5% TFA and incubating for 1 h at RT; this step was repeated for two times total. Extracts were pooled into the same tube with the digestion buffer and dried down on a SpeedVac for 2–4 h. Peptide desalting was carried out by resuspending in 10 µL solvent A (0.1% formic acid in nanopure-diH_2_O) using Pierce 10 µL C18 pipette tips (ThermoScientific, cat. no. 87782) following the manufacturer’s instructions. Briefly, the tip was pre-wetted with 10 µL 50% ACN, followed by equilibration for two times using 10 µL solvent A. Peptides were loaded onto the tip by aspirating and dispensing up to 10 µL of sample several times. The tip with the sample was rinsed two times with 10 µL solvent A, discarding solvent with each step. Peptides were then eluted with solvent B (0.1% formic acid in 80% ACN), followed by drying on a SpeedVac. For each sample, resuspended peptide (12 µL) was transferred into the sureSTART MS Vial (Thermo Fisher Scientific) for LC-MS/MS analysis.

### LC-MS/MS identification of proteins from gel slices

Tryptic peptides were loaded onto a 15 cm C18 capillary column (Thermo PepMap, cat. no. ES75150PN) using a Thermo Vanquish Neo UHPLC system. Peptide separation was performed over a 24 min gradient using solvent B (80% acetonitrile with 0.1% formic acid), beginning at 4%, then increasing to 4.5% over 0.7 min, 5% over 0.3 min, 20% over 13 min, 35% over 6.9 min, and 55% over 0.7 min. A final wash step with 99% solvent B was applied to ensure complete elution. Eluted peptides were analyzed on a Thermo Orbitrap Exploris 240 mass spectrometer. MS1 spectra were acquired at a resolution of 120,000 over a scan range of 350–1,600 *m*/*z*, with an AGC target of 3 × 10⁶. MS/MS spectra were acquired using a topN method to collect the top 20 most abundant peptides in each scan cycle with a resolution of 15,000 and a normalized AGC target of 5 × 10^4^. Higher-energy collisional dissociation (HCD) was performed with a normalized collision energy of 30%.

### Statistical analysis

Thermo raw data files were processed using MSFragger within the FragPipe proteomics pipeline (version 22.0) (28). Peptide quantification was carried out using IonQuant, with the match-between-runs (MBR) feature enabled to enhance data completeness across samples. Database searches were performed with default parameters for label-free proteomics, except for a customized precursor mass tolerance of −10 to +10 ppm. Trypsin was specified as the digestion enzyme, allowing up to two missed cleavages. Carbamidomethylation of cysteine (+57.0215 Da) was set as a fixed modification, and oxidation of methionine (+15.9949 Da) was included as a variable modification. Peptides were required to be 7–50 amino acids in length, with a maximum mass of 5,000 Da. Peptide-spectrum matches (PSMs) were filtered to a 1% false discovery rate (FDR) at both the PSM and protein levels using the Philosopher toolkit. Label-free quantification was performed using IonQuant, based on extracted ion chromatogram (XIC) peak areas. Protein intensities were calculated using the MaxLFQ algorithm, and the resulting normalized MaxLFQ values were used for downstream statistical analysis and group comparisons.

## RESULTS

### HvPat1 and HvPat2 are GNAT family homologs with conserved active site residues and distinct genomic neighborhoods

**Fig 1:**
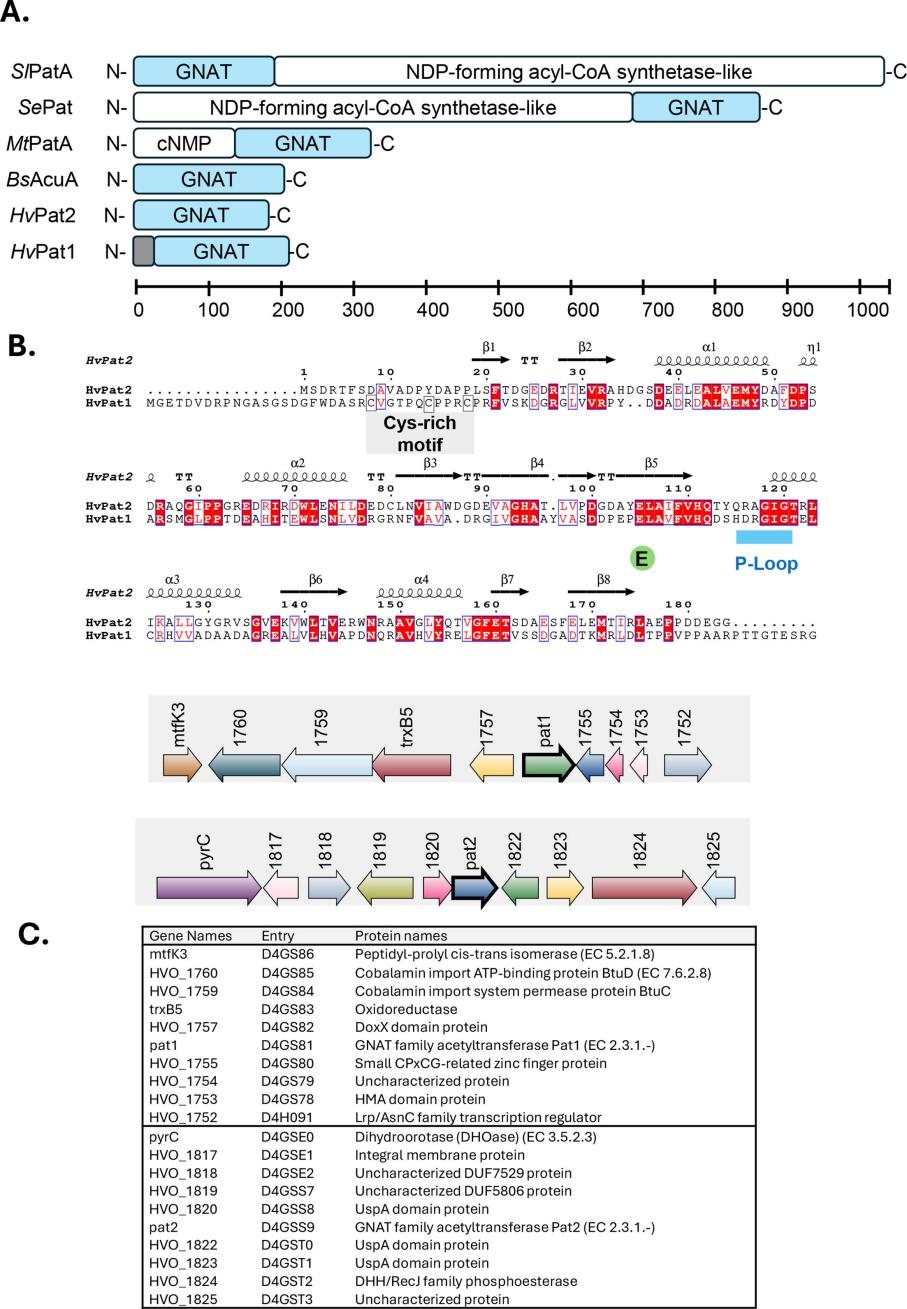
HvPat1 and HvPat2 are GNAT family homologs with conserved active site glutamate and P-loop motif. (**A**) Domain architecture of representative GNAT family proteins. *Sl*, *Streptomyces lividans; Se*, *Salmonella enterica; Mt*, *Mycobacterium tuberculosis; Bs*, *Bacillus subtilis*. Gray box, conserved C-X_5_-C-X_3_-C motif of HvPat1. Modified from reference (31) to include archaeal proteins. (**B**) Amino acid sequence alignment of HvPat1 and HvPat2. Conserved residues, white text and highlighted in red. Functionally similar residues, red text, and boxed in blue. Highlighted: P-loop (blue), conserved CoA binding pocket located between the predicted β5 and α3 regions of HvPat2 (and HvPat1) based on AlphaFold 3D-structural modeling. P-loop plays a crucial role in binding the pyrophosphate group of acetyl-CoA in GNAT family homologs. Glu (E green), conserved active site glutamate that may act as a general base to deprotonate the amino group of the lysine residue of the substrate during catalysis. Cys-rich motif (gray), C-x_5_-C-x_3_-C, conserved among HvPat1 homologs. Sequence alignment performed with Clustal Omega (26). Output processed by ESPript 3.0 (http://espript.ibcp.fr/ESPript/cgi-bin/ESPript.cgi) (27). (**C**) Genomic neighborhood of the *pat1* and *pat2* genes. Gene names are shown above the open reading frames, depicted as arrows, and correspond to the UniProt entry numbers and protein descriptions listed in the table below.

*H. volcanii* (Hv signifies organism of origin) Pat1 and Pat2 are single-domain GNAT family proteins that share 32.4% amino acid sequence identity (Fig. 1A and B). While bacterial GNATs are often fused to regulatory domains like the cNMP and NDP-forming acyl-CoA synthetase-like domains (13, 29–31), this single GNAT domain architecture of HvPat1 and HvPat2 is common among archaeal homologs. A distinguishing feature of HvPat1 and its close homologs is an N-terminal Cys-rich motif (C-x_5_-C-x_3_-C, where x is any amino acid) predicted to play a role in metal ion coordination or redox processes. GNATs, though diverse, share a conserved core including a P-loop that plays a crucial role in binding the pyrophosphate group of acetyl-CoA (13, 29). A P-loop motif is conserved and located between the predicted β5 and α3 regions in HvPat1 and HvPat2 based on AlphaFold 3D-structural modeling. Moreover, HvPat1 and HvPat2 are predicted to be catalytically active, based on the conservation of a glutamate residue (HvPat2 E105 and HvPat1 E119) that could serve as a general base to deprotonate the amino group of the substrate lysine residue and facilitate nucleophilic attack on the acetyl group of acetyl-CoA during the catalytic reaction (32). The HvPat1 and HvPat2 proteins are encoded in distinct genomic regions, with the *pat1* gene linked to oxidative stress response gene homologs, such as those encoding DoxX domain and TrxB5 oxidoreductases, while the *pat2* gene overlaps *hvo_1820*, encoding a UspA domain-containing protein (Fig. 1C).

### Δ*pat2* mutant displays loss of viability and cellular aggregation upon reaching the stationary phase on glycerol compared to glucose

**Fig 2:**
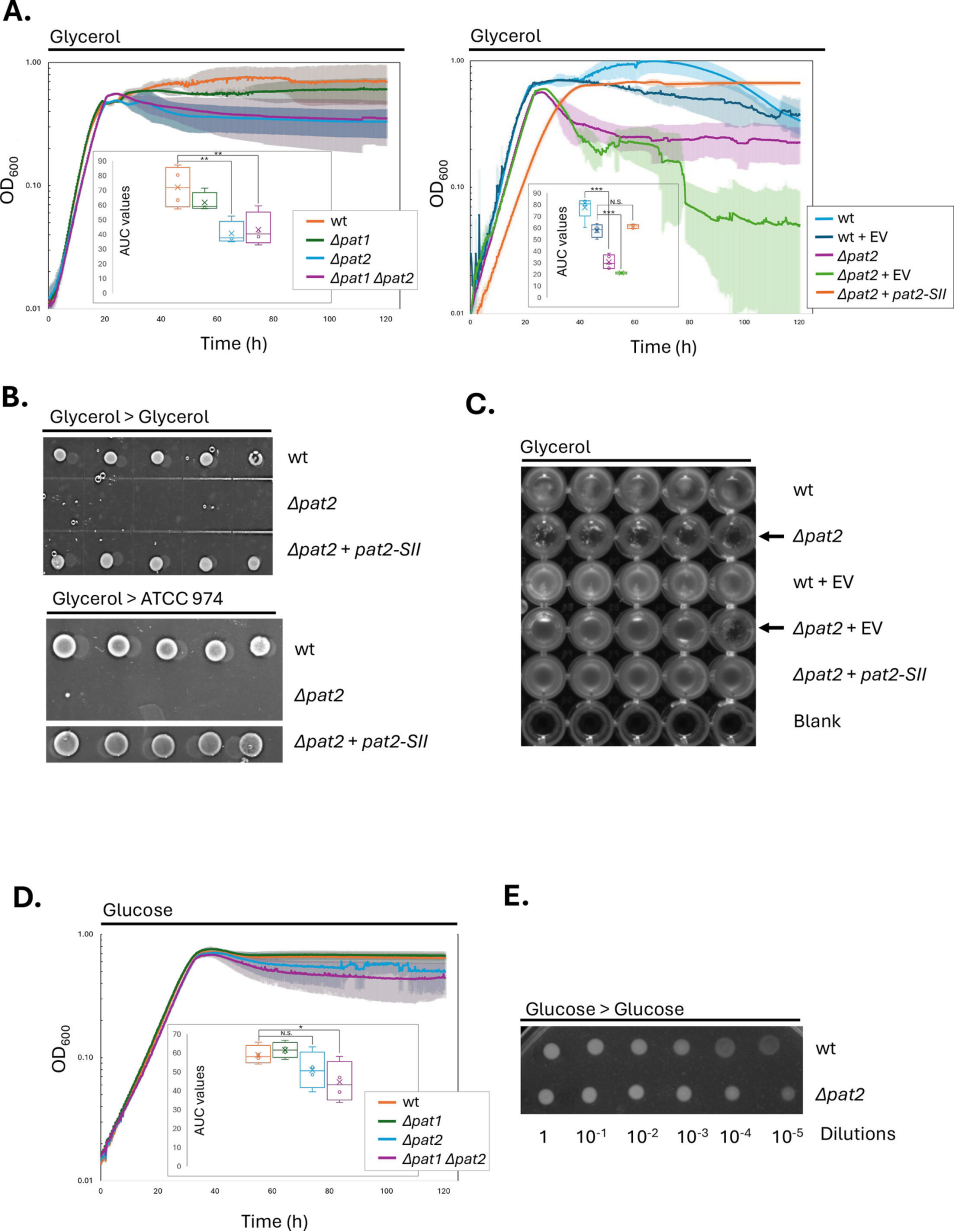
*H. volcanii* Δ*pat2* mutant displays loss of viability and cellular aggregation upon reaching the stationary phase on glycerol compared to glucose. (**A**) Growth defect of the Δ*pat2* mutant on glycerol, as observed by a drop in OD_600_ during the stationary phase. Left, growth curves of wt (H26, orange), ∆*pat1* (JM501, green), ∆*pat2* (JM502, blue), and ∆*pat1* ∆*pat2* (JM504, purple). Right, growth curves of wt (light blue), wt + EV (H26-pJAM202c, dark blue), ∆*pat2* (JM502, purple), ∆*pat2* + EV (JM502-pJAM202c, green), and ∆*pat2 + pat2* SII (JM502-pJAM4017, orange). Strains were grown on glycerol minimal medium. Abbreviations: EV, empty vector. SII, C-terminal StrepII tag. wt, wild type (parent strain). See Materials and Methods for details. Each experiment was performed using at least 3 replicates, and then the average OD_600_ for each strain/condition at each time point was taken. The blank value for each medium type was subtracted from the averaged values and graphed on the log scale. Inset graphs show area under the curve (AUC) values for each strain. * *P*-value ≤ 0.05, ** *P*-value < 0.01, *** *P*-value < 0.0001, N.S. no significant difference. Experiments were performed at least in duplicate and found to be reproducible. (**B**) Loss of viability of the ∆*pat2* mutant after growth to the stationary phase on glycerol. Cells grown to the stationary phase on glycerol minimal medium (120 h, panel A) were serially diluted and spotted onto agar plates of glycerol minimal medium or ATCC 974 rich medium, as indicated. The plates were imaged after 1-week incubation at 42°C. Compared to wt (parent H26) and ∆*pat2* expressing *pat2-SII* from a plasmid (JM502-pJAM4017), the ∆*pat2* mutant was found to be non-viable. (**C**) Δ*pat2* mutant aggregates after growth on glycerol. Image of the strains in the microtiter plates immediately following the 120 h incubation with aeration at 42°C in glycerol minimal medium. Aggregated cells indicated by arrow. Images acquired using the nucleic acid setting with an iBright imaging system. (**D**) On glucose, the Δ*pat1* and Δ*pat2* mutants exhibit growth patterns that are largely similar to wild type, as indicated by OD_600_ growth curves. wt (H26, orange), ∆*pat1* (JM501, green), ∆*pat2* (JM502, blue), and ∆*pat1* ∆*pat2* (JM504, purple). (**E**) ∆*pat2* mutant does not display loss of viability after growth to the stationary phase on glucose. Cells grown to the stationary phase on glucose minimal medium (120 h, panel D) were serially diluted and spotted onto agar plates of glucose minimal medium.

Lysine acetylation is a well-known regulatory mechanism that enables cells to rapidly and dynamically adapt to shifts in carbon source utilization (33, 34). We reasoned that whether HvPat1 and HvPat2 mediate the lysine acetylation of key enzymes involved in carbon metabolism, then deletion of *pat1* and/or *pat2* may lead to growth phenotypes dependent upon the available carbon source. To investigate this possibility, we analyzed the growth and stationary-phase survival of *H. volcanii* “wild-type” (wt, parent), ∆*pat1*, ∆*pat2*, and ∆*pat1* ∆*pat2* strains along with a ∆*pat2* mutant complemented by ectopic expression of *pat2+* using glycerol and glucose as carbon sources (Fig. 2, Fig. S1). Growth was monitored by measuring optical density at 600  nm (OD_600_) over 120 h using a microtiter plate assay. Stationary phase survival was assessed by spot plating the final cultures in serial dilutions onto agar plates. The cultures were also visually inspected in the microtiter plate immediately after the 120 h assay. Distinct phenotypes emerged when cells were grown on glycerol compared to glucose. Specifically, on glycerol, the ∆*pat2* and ∆*pat1* ∆*pat2* mutant strains demonstrated a pronounced drop in OD_600_ during the stationary phase that was not observed in the wt or ∆*pat1* mutant strains (Fig. 2A, left). This phenotype was rescued by ectopic expression of *pat2-SII* (a StrepII-tagged version of *pat2*), confirming the defect was due to the *pat2* deficiency (Fig. 2A, right). Further analysis revealed the ∆*pat2* mutant strains lost viability after extended growth on glycerol as shown by spot plating (Fig. 2B). Once this loss of viability occurred in glycerol, it could not be overcome by shifting the ∆*pat2* mutant cells to a rich medium (e.g.*,* ATCC 974) (Fig. 2B). Visual inspection of the microtiter plates showed that the loss of viability of the *∆pat2* mutant was correlated with cellular aggregation (Fig. 2C). In contrast, all strains showed somewhat comparable growth and survival on glucose, with similar entry into exponential and stationary phases and no notable loss of viability (Fig. 2D and E). Together, these results demonstrate a carbon source-dependent phenotype associated with *pat2* deficiency: when grown on glycerol, ∆*pat2* mutants form aggregates and lose viability after reaching the stationary phase, a defect not observed with glucose as the carbon source.

### HvPat2 purifies as a monomer

**Fig 3:**
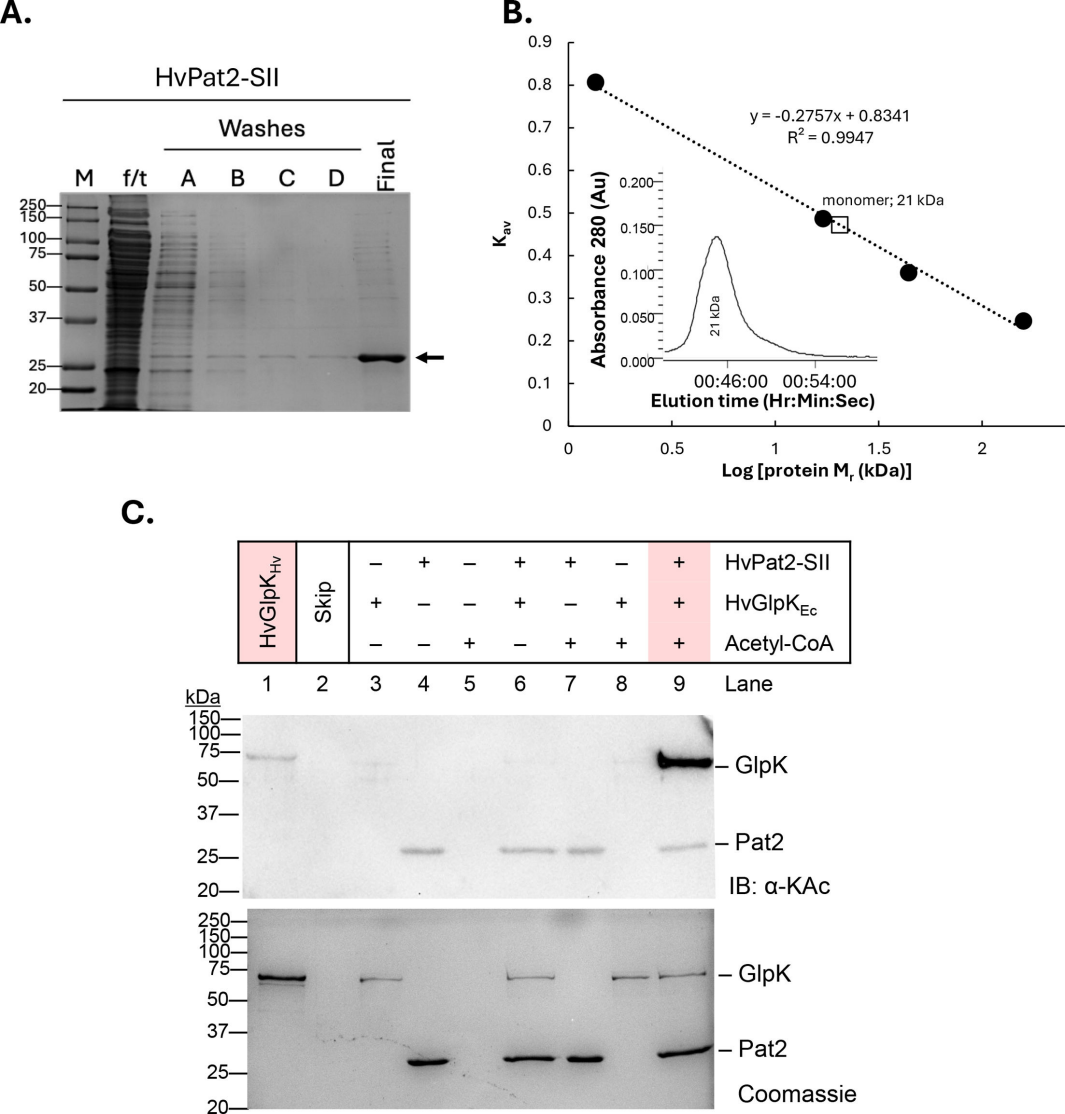
Purified HvPat2 catalyzes the lysine acetylation of glycerol kinase (HvGlpK). (**A**) HvPat2-SII was enriched by StrepTactin affinity chromatography. HvPat2-SII was purified from 3 to 3.5 g (wet weight) cell pellet of H26 ∆*pat2*-pJAM4554 expressing the SII-tagged protein in *trans*. Cells were cultured to the stationary phase in Hv-CA+ medium. Cells were lysed with a French press. Cell lysate was clarified and applied to StrepTactin resin. Flowthrough (f/t) was collected, resin was washed (lanes A and B), and f/t was reapplied to the column for a total of four washes (lanes A through D). HvPat2-SII was eluted with binding buffer supplemented with 1 mM desthiobiotin. Fractions of f/t (1 µL), first set of washes (5 µL), and final eluted protein (5 µL) were separated on 12% SDS-PAGE. Protein gels were stained with Coomassie blue for 30 min, briefly rinsed with diH_2_O, then destained with fresh diH_2_O (see Materials and Methods for details). (**B**) Size exclusion chromatography demonstrates that HvPat2-SII purifies as a monomer with a molecular mass of 21 kDa. (**C**) HvPat2 catalyzes the lysine acetylation of HvGlpK. Reactions included HvPat2, acetyl-CoA, and HvGlpK purified from recombinant *E. coli* (_EC_) as indicated. Endogenously acetylated glycerol kinase purified from *H. volcanii* (HvGlpK_Hv_) served as a positive control. Lysine acetylation reactions were assembled *in vitro* using purified HvPat2-SII (6 µM) as enzyme, His-HvGlpK_Ec_ (2 µM) as protein substrate, and acetyl-CoA (0.2 mM) as the acetyl group donor (Materials and Methods). Reactions were incubated at 37°C for 3 h. Products were resolved by 12% SDS-PAGE and analyzed by immunoblotting using anti-acetyl-lysine (IB: αKac; top image) and Coomassie Blue stain (bottom, to control for protein loading).

To examine catalytic function, the HvPat2 protein was expressed with a C-terminal StrepII tag (HvPat2-SII) and purified from *H. volcanii* by StrepTactin affinity chromatography. SDS-PAGE analysis demonstrated HvPat2-SII could be purified to relative homogeneity as a protein band that migrated at 25 kDa (Fig. 3A and B). Further purification using size exclusion chromatography (SEC) revealed elution profiles consistent with HvPat2 adopting a native molecular mass corresponding to the monomeric form (Fig. 3B inlay). For reference, HvPat2-SII-tagged proteins are 196 amino acids in length with the theoretical isoelectric point (pI) calculated to be 4.2, where the estimated molecular mass is 21,636 Da. No additional protein partners were observed to co-purify with HvPat2-SII.

### HvPat2 catalyzes the lysine acetylation of HvGlpK

We reasoned that whether HvPat2 is essential for survival on glycerol (vs. glucose) as a carbon source, then it may act on key enzymes of glycerol metabolism. Given its central role in glycerol metabolism, glycerol kinase (HvGlpK) emerged as a strong candidate for further analysis. HvGlpK catalyzes the phosphorylation of glycerol to glycerol-3-phosphate, a key metabolic step in glycerol metabolism, and is essential for growth on glycerol (35, 36). Moreover, our previous studies showed that HvGlpK is acetylated at K153 in cells grown on glycerol (37), suggesting a potential regulatory mechanism involving lysine acetylation. The single K153 residue found modified suggested that the acetylation of HvGlpK may be catalyzed by a GNAT family enzyme instead of occurring non-enzymatically through reactions with acetyl-CoA or acetyl-P that can lead to the acetylation of multiple lysine residues (7, 38). To isolate a non-acetylated form of HvGlpK for use as a substrate in our assays, the *H. volcanii glpK* (*hvo_1541*) gene was expressed with an N-terminal His tag in *E. coli*. The recombinantly expressed His-HvGlpK_Ec_ protein was then purified by Ni^2+^-affinity chromatography (Fig. S2) followed by SEC and found to be in a non-acetylated state based on immunoblotting with anti-acetyllysine antibodies (IB: αKac) (Fig. 3C*,* lane 3). Thus, His-HvGlpK_Ec_ could be examined as a potential substrate of HvPat2-SII by *in vitro* assay using acetyl-CoA as an acetyl group donor (Fig. 3C). After incubation of purified components, the reaction products were resolved by SDS-PAGE and analyzed by IB: αKac. In the absence of HvPat2, His-HvGlpK_Ec_ did not react with acetyl-CoA (lane 8), indicating that this acetyl-group donor does not react with the glycerol kinase through a non-enzymatic mechanism. Additionally, no signal was detected that would indicate lysine acetylation of the substrate when HvPat2 and His-HvGlpK_Ec_ were incubated without acetyl-CoA (lane 6), meaning an acetyl-group donor is required for lysine acetylation of His-HvGlpK_Ec_. By contrast, when His-HvGlpK_Ec_ was incubated with both HvPat2 and acetyl-CoA, a strong signal for lysine acetylation of this substrate was detected (lane 9). This acetylated form of HvGlpK migrated similarly to the lysine acetylated form of HvGlpK purified from *H. volcanii* (*Hv*GlpK_Hv_) (lane 1). A weak signal of lysine acetylation was also detected that migrated with HvPat2-SII (lanes 4, 6, 7*,* and 9), irrespective of the addition of acetyl-CoA to the reaction, suggesting that HvPat2-SII is purified from *H. volcanii* in a lysine acetylated form. This finding is consistent with our previous high-throughput proteomic analysis that found HvPat2 K125 to be acetylated in glycerol-grown cells (37). Overall, these results suggest that HvGlpK is a substrate of HvPat2 and that HvPat2 functions as a catalytically active lysine acetyltransferase of protein substrates.

### Key residues of HvPat2 predicted to be involved in acetyl-CoA binding and catalysis identified by *in silico* modeling

**Fig 4:**
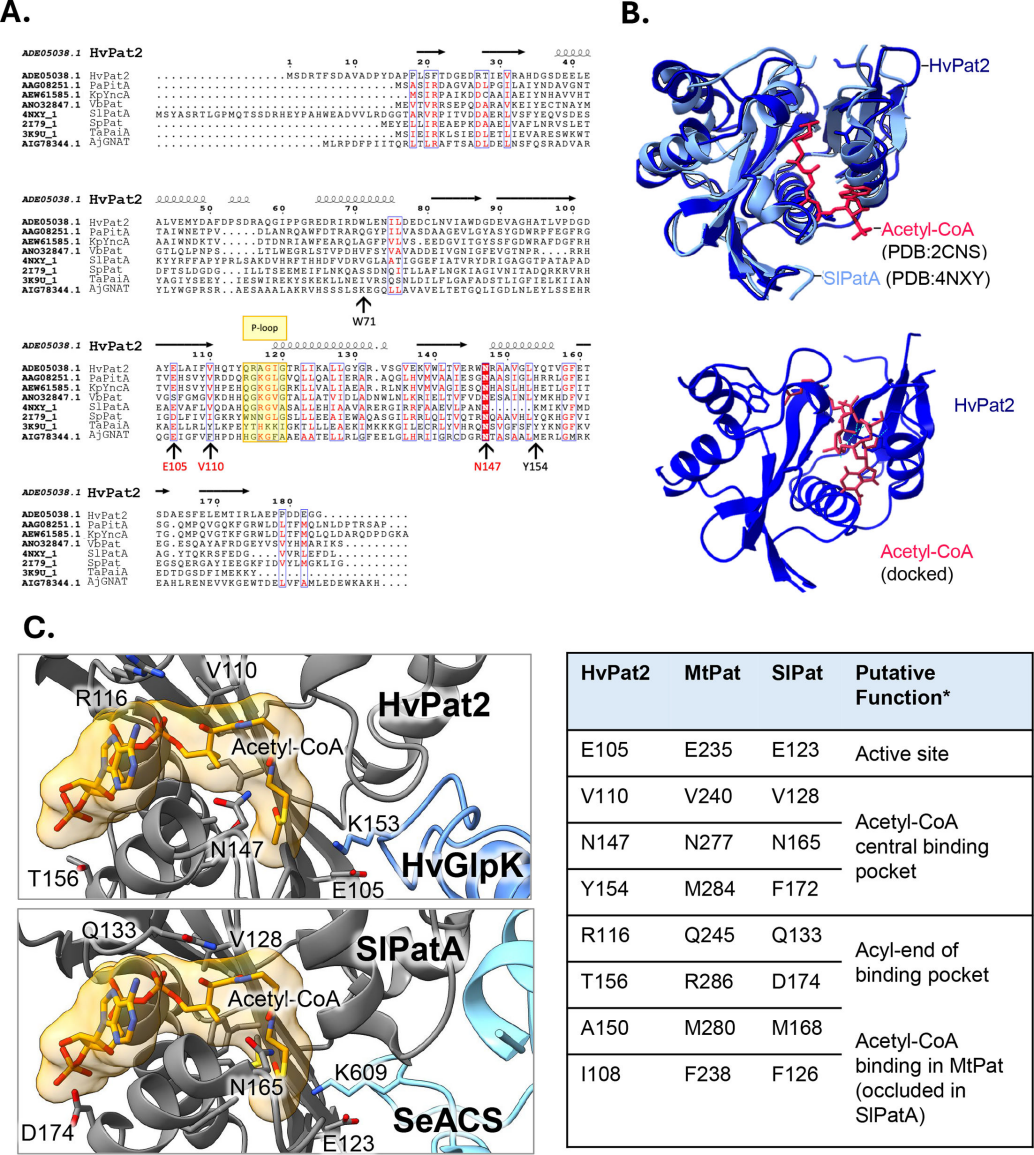
*Hv*Pat2 secondary structure prediction suggests Glu105, Val110, Asn147, and Tyr154 to be involved in A-CoA binding. (**A**) Sequence conservation and secondary structure of HvPat2-related GNATs. GNAT-related lysine acetyltransferases act on a variety of substrates and share a distinct ɑ/β-fold, the conserved acetyl-coenzyme A binding domain known as the pyrophosphate binding loop (P-loop), and a catalytic Glu residue. FoldSeek was used to identify proteins with structures and sequences that are like the predicted HvPat2 structure (ADE05038). Selected sequences were aligned using ClustalW (.aln file) including (PDB or GenBank accession number): *S. lividans* SlPatA (PDB: 4NXY), *Pseudomonas aeruginosa* PaPitA (AAG08251.1), *Klebsiella pneumoniae* KpYncA (AEW61585.1), *Vibrio breoganii* VbPat (ANO32847.1), *Streptococcus pneumoniae* SpPat (PDB: 2I79), *Thermoplasma acidophilum* TaPaiA (PDB: 3K9U), *Haloferax volcanii* HvPat2 (ADE05038), and *Amycolatopsis japonica* AjGNAT (AIG78344.1). Secondary structure of the aligned sequences was predicted against SlPatA (PDB: 4NXY). Residues in red are semi-conserved, and those highlighted in red are conserved. P-loops are boxed in yellow. Alignment was made using ESPript 3.0 (27). (**B**) 3D-structural modeling of HvPat2 bound to acetyl-CoA. (Top) ChimeraX was used to superimpose the predicted *Hv*Pat2 structure (royal blue) with the crystal structure of *SI*PatA (light blue*),* and the crystal structure of acetyl-CoA (from PDB: 2CNS). (Bottom) The crystal structure of acetyl-CoA (PDB: 2CNS) was docked with *Hv*Pat2. Acetyl-CoA binding is consistent with superimposed structure and implicates Trp71, Glu105, Val110, Asn147, and Tyr154 in forming H-bonds with acetyl-CoA. This conformation represents the highest affinity docking position possible between *Hv*Pat2 and acetyl-CoA (−8.1 kcal/mol; PyRx). (**C**) Docking of HvGlpK with HvPat2 bound with acetyl-CoA. AlphaFold model of HvPat2 bound to HvGlpK. While the ipTM score of 0.28 for this model is low, the site of HvGlpK acetylation (K153) (37) is in close proximity to the proposed HvPat2 active site residues. X-ray crystal structures of *Mycobacterium tuberculosis* MtPat bound to acetyl-CoA (PDB: 4avb) and *Streptomyces lividans* SlPat bound to *Salmonella enterica* SeACS (acetyl-CoA synthetase) (PDB: 4u5y) were used for comparison. Contacts of HvPat2 E105, V110, N147, and Y154 residues are highlighted. 3D structural comparison of key residues of the bacterial Pat enzymes to HvPat2 is noted on the right.

All GNATs form a characteristic GNAT fold that encompasses the pyrophosphate-loop, or P-loop, for acetyl-CoA binding (29). Given that there is no currently available crystal structure of HvPat2, we decided to focus our initial efforts on pinpointing the catalytic residues involved in or around the Pat2 active site by docking acetyl-CoA with the predicted Pat2 model (Fig. 4). First, Foldseek was used to identify proteins across all domains of life that are predicted to have high structural homology with the predicted AlphaFold structure of HvPat2 (ADE05038.1) (Fig. 4A). The search yielded both solved and predicted GNAT proteins and those with the highest structural homology (PDB: 4NXY and 2I79 and GenBank: AAG08251.1, AEW61585.1, and ANO32847.1) were chosen for further comparisons. Next, the primary sequence of each protein was aligned to the HvPat2 sequence based on sequence similarity using Clustal Omega (26).

Despite having overall low sequence homology, the secondary structure formed by GNAT-related enzymes is highly conserved across species and consists of four conserved motifs (C, D, A, and B) that form the GNAT-fold. These motifs are composed of a mix of α/β-folds where there are six or seven β-strands and four helical strands (10). To determine whether this trend could be observed in our data set, the multiple sequence alignment file was overlaid with the HvPat2 secondary structure predicted by AlphaFold (39) using ESPript 3.0 (27) (Fig. 4A). The results suggest that the predicted secondary structures formed by HvPat2 match the universally conserved GNAT fold identified in previous studies (10). In addition, the highly conserved catalytic Glu residue (E105 in HvPat2) and a plausible P-loop (Q115-G120) were observed, among other semi-conserved residues, including V110 and the absolutely conserved N147 residue (31, 40). Next, the AlphaFold-generated 3D-structural model of HvPat2 was superimposed with the X-ray crystal structure of *S. lividans* PatA (SiPatA) bound to acetyl-CoA to identify the putative acetyl-CoA binding pocket of HvPat2. To propose specific residues involved with acetyl-CoA binding, ChimeraX was used in conjunction with PyRx molecular docking software (Fig. 4B). The predicted 3D structure of HvPat2 was prepared for docking by removing water molecules, assigning partial charges, and adding hydrogens using ChimeraX dock prep software. Using the prepared HvPat2 and a crystal structure of acetyl-CoA (PDB: 4CNS), the most energetically efficient conformation in which acetyl-CoA binds to the enzyme within the specified binding cleft was predicted using PyRx. The PyRx molecular docking tool runs on Autodock Vina and returns the most likely conformation with the lowest binding energy. Docking results implicated five *Hv*Pat2 residues that participate in hydrogen bonding with acetyl-CoA: Trp71, Glu105, Val110, Asn147, and Tyr154. Secondary structural analysis revealed that four of the five residues are positioned close to the proposed P-loop and β-bulge regions of the enzyme (E105, V110, N147, and Y154). With this in mind, we then modeled HvPat2 docked with acetyl-CoA and HvGlpK (Fig. 4C). These results support prior docking models and suggest that Val110, Asn147, and Tyr154 residues appear to be involved in the central pocket for binding acetyl-CoA, while E105 may function as a catalytic residue in the active site.

### Purification of Pat2 variants and the association of HVO_2384

**Fig 5:**
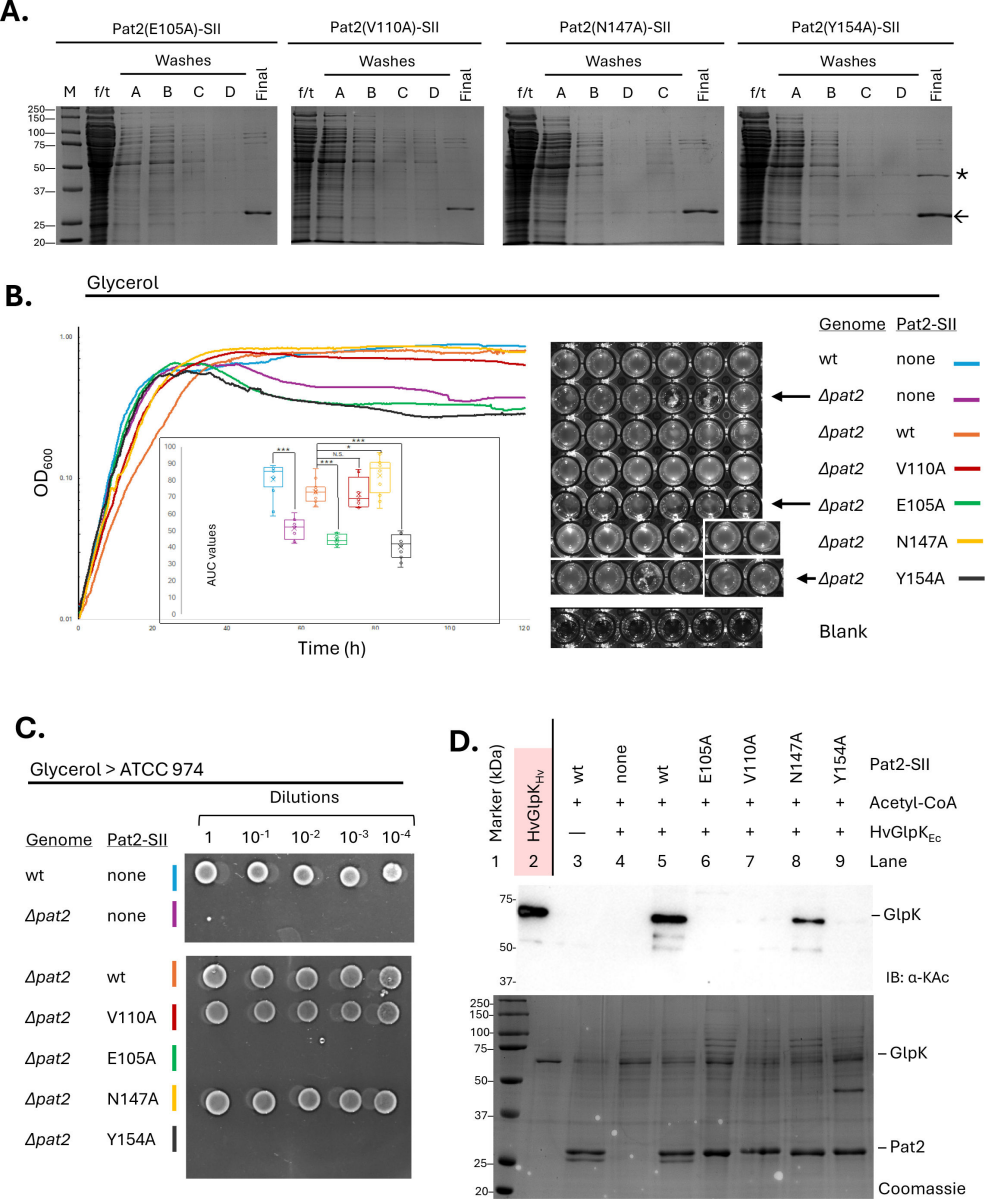
HvPat2 residues identified by *in silico* 3D modeling were found to be important for catalyzing lysine acetylation of HvGlpK by *in vitro* assay. (**A**) Purification of HvPat2-SII variant proteins from *H. volcanii*. Strains for purification were based on H26 ∆*pat2* carrying plasmids pJAM4555 (HvPat2-SII E105A), pJAM4556 (HvPat2-SII V110A), pJAM4557 (HvPat2-SII N147A), or pJAM4558 (HvPat2-SII Y154A). Cells were cultured in ATCC974. Recombinant protein expression was induced by adding 2 mM L-Trp when cultures reached an OD_600_ 0.6–0.8. Cultures were continued overnight and then harvested by centrifugation. Cell pellets (1.5 g) were lysed using a French Press. StrepII-tagged proteins (-SII) were purified by applying the clarified lysate to columns packed with StrepTactin resin. Flow-through (f/t) was collected and reapplied to the column for a second set of washes. StrepII-tagged proteins were eluted with binding buffer supplemented with 1 mM desthiobiotin. Fractions of f/t (1 µL), first set of washes (5 µL), and final eluted protein (5 µL) were separated on 12% SDS-PAGE. Protein gels were stained with Coomassie blue for 30 min, briefly rinsed with diH_2_O, then destained with fresh diH_2_O overnight. (**B**) E105 and Y154 appear important for HvPat2 activity during glycerol metabolism. Growth curves (left) of parent H26 (wt, light blue), ∆*pat2* (purple), and ∆*pat2* transformed with plasmids expressing Pat2-SII wild-type (wt, orange) or site-directed variants E105A (green), V110A (red), N147A (yellow), or Y154A (black). Strains were monitored for growth on glycerol minimal medium by OD₆₀₀ measurements in a microtiter plate assay (left). Inset graphs show the area under the curves for each strain compared to wt. * *P*-value ≤ 0.05, ** *P*-value < 0.01, *** *P*-value < 0.0001, N.S. no significant difference. Image of microtiter plate (right) immediately following growth curve demonstrates cells settled at the bottom of the well and depicts the “clumping” phenotype (arrow). Blank, no inoculum control. See Materials and Methods for details. (**C**) HvPat2 E105 and Y154 are important for overcoming the loss of viability of the ∆*pat2* mutant strains after growth on glycerol. Cells from the glycerol growth curve (120 h time point) were serially diluted, spotted onto ATCC974-rich medium plates, and incubated for 1 week at 42°C. The results demonstrate that the aggregated cells at the bottom of wells are no longer viable when diluted onto agar plates. Similar results are observed on GMM plates. Portions of this figure were from the same experiment as Fig. 2B and reused to highlight the comparison between the wild type and the *pat2* mutant. (**D**) All four residues (E105, Y154, V110, and N147), predicted to be involved in catalysis or acetyl-CoA binding, were found to be important for full HvPat2 activity in mediating the lysine acetylation of HvGlpK *in vitro*. Lysine acetylation reactions with HvPat2 variants were performed *in vitro* as previously described. Products were resolved by 12% SDS-PAGE and stained with Coomassie or detected using anti-acetyl lysine antibodies.

To investigate the functional roles of specific HvPat2 residues, we used a reduced-recycle PCR (rrPCR)-based site-directed mutagenesis approach (25) to individually substitute E105, V110, N147, and Y154 with alanine. The mutagenesis was performed using plasmid pJAM4017, which expresses the HvPat2-SII fusion protein, as the template. The modified plasmids were transformed into the ∆*pat2* mutant, and the HvPat2 variant proteins were expressed and purified by StrepTactin affinity chromatography as described for HvPat2-SII. By this approach, each of the HvPat2 variant proteins was found to purify as a band that migrated around 25 kDa when resolved by SDS-PAGE (Fig. 5A). HvPat2 Y154A fractions were found to include an additional protein migrating around 50 kDa. Analysis of this co-purifying protein by LC-MS/MS revealed it to be HVO_2384 (UniProt ID: D4GWN2), which had a theoretical molecular mass of 42,521 Da and pI of 4.87, consistent with SDS-PAGE migration of this acidic protein. HVO_2384 harbors tandem cystathionine β-synthase (CBS; or Bateman) domains structurally related to those of the dual ribosomal subunit inhibitor (Dri, Pcal_1808), shown to bind ribosomes and inhibit translation *in vitro* (41). In contrast to Dri of arCOG00601, HVO_2384 is of the more broadly distributed arCOG00601 and has an additional C-terminal domain related to the ribosome hibernation factor (RHF)-like superfamily (IPR036567) (42), implying that it too may associate with and inhibit activity of ribosomes but act by a different mechanism. Given that CBS domains often bind adenosine-containing metabolites and serve as energy-sensing regulatory modules (43), it is plausible that HVO_2384 serves as a link between Pat2-mediated lysine acetylation, cellular energy status, and translational repression.

### Key residues important for HvPat2 function *in vivo*

As all the HvPat2 variant proteins were found to be expressed from the plasmids and could be purified from the *H. volcanii* ∆*pat2* mutant, we next examined the growth and viability of these strains on glycerol. As previously observed controls, the ∆*pat2* mutant demonstrated a drop in OD_600_ during the stationary phase, while the parent and ∆*pat2* mutant strain expressing *pat2+* (*pat2-SII* wt) did not (Fig. 5B**,** left). In contrast to HvPat2 V110A and N147A, the OD_600_ of the strains expressing HvPat2 E105A and Y154A dropped in the stationary phase, similar to what was observed for the ∆*pat2* mutant. Further analysis revealed a correlation between those strains that displayed a drop in OD_600_ and clumping at the bottom of the wells in the 96-well plate in a non-uniform manner (Fig. 5B**,** right). Additionally, the strains that demonstrated this clumping phenotype were no longer viable when transferred from the glycerol minimal medium (GMM) stationary phase cultures in the 96-well microtiter plate to either rich medium (ATCC974) or GMM agar plates (Fig. 5C). These results suggest that HvPat2 E105A and Y154A are inactive, while the V110A and N147A variants can complement the ∆*pat2* mutant for growth.

### Key residues important for HvPat2 function *in vitro*

To further investigate residues predicted by *in silico* analysis to be important for HvPat2 acetyl-CoA binding or catalytic activity, the lysine acetylation activity of the purified HvPat2-SII variants was assessed by *in vitro* assay using acetyl-CoA and HvGlpK_Ec_ as substrates (Fig. 5D). In contrast to the robust activity of HvPat2-SII wt, no catalytic activity was detected for the E105A and Y154A variants. The V110A variant showed minimal activity, while N147A exhibited reduced activity. Moreover, the HVO_2384 protein that co-purified with HvPat2 Y154A was not found to be lysine-acetylated based on the anti-Kac immunoblot assay (Fig. 5D, lane 9). Our previous work found HVO_2384 is acetylated at K311 and K313 in glycerol-grown cells based on LC-MS/MS analysis of tryptic peptides enriched using an anti-acetyllysine antibody (37). This distinction prompted us to examine whether the “wild-type” HvPat2-SII could use acetyl-CoA to mediate the lysine acetylation of HVO_2384 when HVO_2384 is purified in complex with the inactive variant HvPat2 Y154A. However, no lysine acetylation of HVO_2384 was detected, suggesting that when bound to the inactive form of HvPat2, HVO_2384 is not accessible or properly positioned for acetylation. Overall, the *in vitro* activity of the HvPat2 variants is consistent with the *in vivo* results and indicates that E105 and Y154 are critical for enzymatic function.

## DISCUSSION

This study reveals that deletion of *pat2*, but not *pat1*, leads to impaired survival of *H. volcanii*, characterized by a carbon source-dependent clumping of cells that results in a dramatic reduction in viability during early stationary phase on glycerol. Consistent with the ∆*pat2* mutation being responsible for this phenotype, this growth defect is alleviated by restoring expression of *pat2* in *trans*. Through *in vitro* reconstitution, we show that HvPat2 can catalyze the lysine acetylation of glycerol kinase (HvGlpK). Given that deletion of *pat2* impairs surival on glycerol but not glucose (this study) and that HvGlpK is essential for glycerol metabolism (36), we suggest that HvPat2-mediated lysine acetylation positively regulates the structure and/or function of HvGlpK and is critical for *H. volcanii* survival on glycerol minimal medium. Supporting the idea that Pat2 is a positive regulator of GlpK that promotes its activity and stability, HvGlpK is reported to be more active when lysine acetylated and is undetectable in the ∆*pat2* mutant compared to wild type when grown on glucose or glycerol (44).

To better understand HvPat2 function, *in silico* 3D structural modeling was used to guide site-directed mutagenesis, followed by analysis of the resulting HvPat2 variants. Four residues (E105, V110, N147, and Y154) were targeted for alanine-scanning mutagenesis based on their predicted role in catalysis and/or acetyl-CoA binding. Of these, the E105A and Y154A variants were found to abolish HvPat2-mediated lysine acetylation of HvGlpK and impair survival on glycerol. Substitutions at V110 and N147 also reduced lysine acetylation *in vitro* but had a milder impact *in vivo*, suggesting that even low or undetectable HvPat2 activity, as measured *in vitro*, may be sufficient to maintain cell viability on glycerol. The HvPat2 Y154A variant was found to co-purify with an additional protein, suggesting it may have a higher affinity for certain proteins than the HvPat2 “wild type” and other variants. While the identity of the protein that co-purifies with HvPat2 Y154A remains to be determined, it does not appear to be lysine acetylated. One could speculate that it may function as a substrate for HvPat2, given that the Y154A mutant does not display any catalytic activity. Alternatively, if the unknown protein is not a HvPat2 substrate, it may function as a regulator of HvPat2 activity.

While most KATs in the GNAT family differ in sequence similarity and substrate specificity, they typically rely on a sequential or ping-pong catalytic mechanism (34). The ping-pong mechanism requires either a cysteine or serine to be properly oriented in the KAT active site, whereas a Glu or Asp is positioned in the active site for KATs that function through a sequential mechanism. Through this study, we can speculate that the E105 residue is positioned to act as a general base for the deprotonation of the targeted lysine residue. Through a nucleophilic attack, the lysine residue then acts on acetyl-CoA to transfer the thioester carbonyl carbon onto itself.

**Fig 6:**
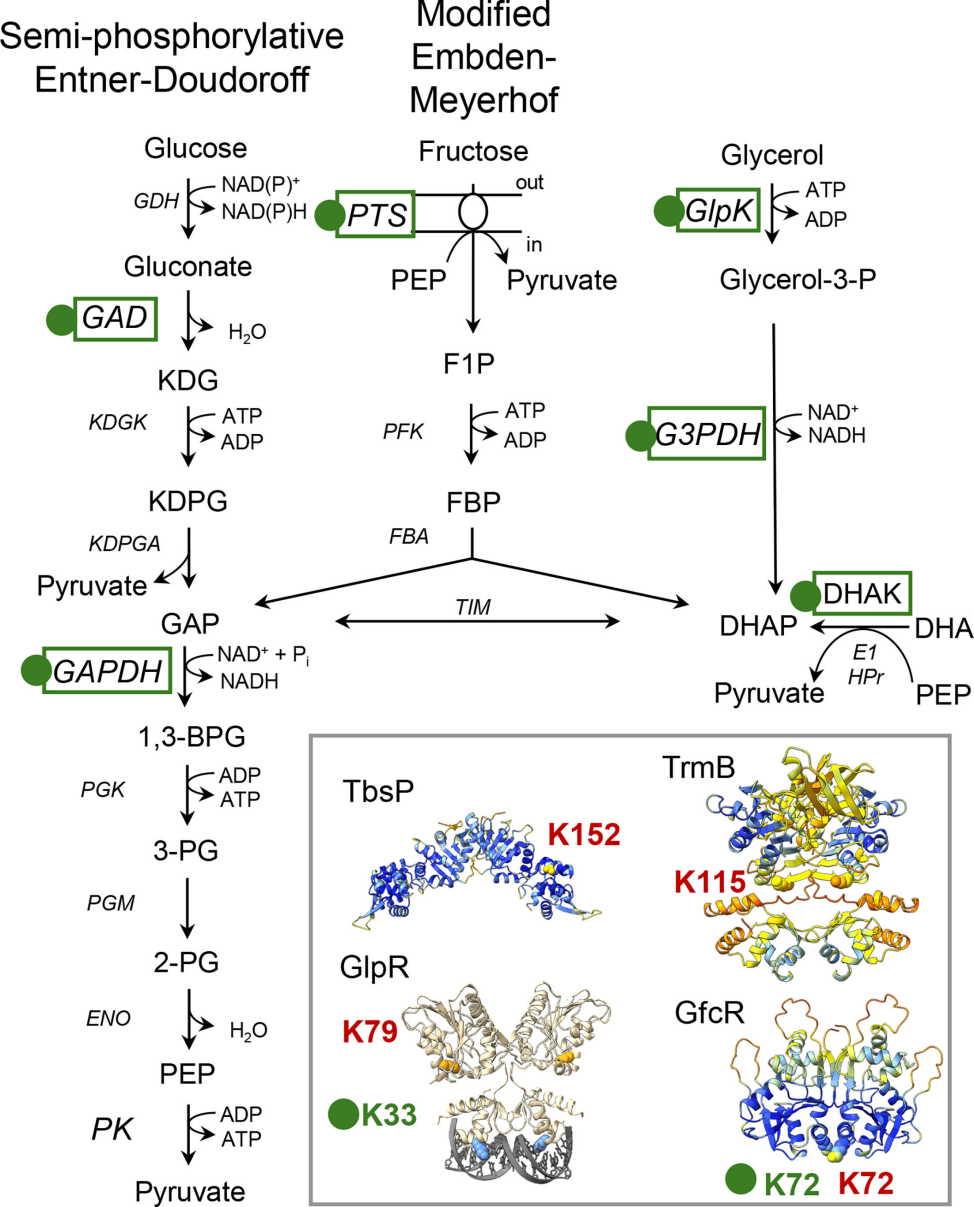
Lysine acetylation of enzymes and transcription factors associated with central carbon metabolism in *H. volcanii*. Kac, lysine acetylation. Green dot, proteins with Kac sites identified in glycerol-grown *H. volcanii* based on a previous study (37). Red, Kac sites of *H. mediterranei* TF homologs identified in glucose-YPC grown cells (50). AlphaFold models of transcription factors associated with central carbon metabolism with Kac sites: TbsP and TrmB (51), GfcR (52), and GlpR (53–55). Metabolites: KDG, 2-keto-3-deoxy-gluconate; KDGP, 2-keto-3-deoxygluconate 6-phosphate; GAP, glyceraldehyde 3-phosphate; 1,3-BPG, 1,3-bisphosphoglycerate; 2 PG and 3 PG, 2- and 3-phosphoglycerate; PEP, phosphoenolpyruvate; F1P, fructose-1-phosphate, FBP, fructose bisphosphate; DHAP, dihydroxyacetone phosphate; DHA, dihydroxyacetone. Enzymes: GDH, glucose dehydrogenase; GAD, gluconate dehydratase; KDGK, KDG kinase; KDGPA, KDGP aldolase; GAPDH, GAP dehydrogenase; PGK, phosphoglycerate kinase; PGM, phosphoglycerate mutase; ENO, enolase; PK, pyruvate kinase; PTS, phosphotransferase system (56), PFK, phosphofructokinase; FBA, fructose bisphosphate aldolase; TIM, triose phosphate isomerase; GlpK, glycerol kinase; G3PDH, glycerol-3-phosphate dehydrogenase; DHAK, dihydroxyacetone kinase (linked to PTS-like EI and HPr phosphorylation cascade) (57, 58). TCA cycle enzymes are all Kac modified on glycerol.

Lysine acetylation is suggested to play a central role in regulating carbon metabolism in *H. volcanii*, potentially through HvPat2-mediated modification of key enzymes like glycerol kinase (HvGlpK). Our prior proteomic analysis of glycerol-grown cells reveals extensive lysine acetylation across proteins involved in central carbon metabolism, including HvGlpK, one of the most highly acetylated enzymes under these conditions (Fig. 6) (37). HvGlpK is essential for glycerol catabolism and lies at a critical metabolic junction, converting glycerol to glycerol-3-phosphate, which feeds into central metabolic pathways (36). While *H. volcanii* can metabolize multiple carbon sources, including simultaneous use of glycerol and fructose or sequential use of glycerol followed by glucose in diauxic growth (36), our findings indicate that the lysine acetyltransferase HvPat2 may be critical for regulating this metabolic flexibility. Δ*pat2* mutants exhibit a severe growth defect specifically on glycerol, but not glucose, suggesting that HvPat2-mediated acetylation is essential for glycerol metabolism. This phenotype likely stems from disrupted acetylation of HvGlpK, implicating post-translational control by HvPat2 as a key determinant of carbon source utilization. Unlike bacterial systems where GlpK is regulated allosterically by fructose bisphosphate and phosphotransferase system (PTS) proteins (45, 46), *H. volcanii* appears to rely on lysine acetylation for fine-tuning GlpK activity, reflecting its distinct metabolic architecture as a heterotrophic, facultatively anaerobic archaeon capable of using a range of electron acceptors (47–49). Together, these data support a model in which HvPat2 modulates central carbon flux by acetylating HvGlpK, thereby influencing the organism’s ability to adapt to different carbon sources, particularly under glycerol-rich conditions.

Our findings expand the understanding of lysine acetylation as a regulatory mechanism in central carbon metabolism. This form of post-translational modification, while understudied in the regulation of archaeal metabolism, is well established in bacterial and eukaryotic metabolic regulation (34, 59). Acetyl-CoA and acetyl-P are central metabolic intermediates that serve as substrates of lysine acetylation (9, 60, 61) and reflect the cellular energy status, residing at the central node between anabolism and catabolism (62). Redox status is also linked to lysine acetylation, as coenzyme A (HS-CoA), a product of acetyl-CoA-dependent lysine acetylation, can serve as a protective thiol in antioxidant defense systems (63). Moreover, sirtuins consume NAD^+^, a key redox-signaling molecule, during the deacetylation reaction (64).

Together in this study, we have identified HvPat2 to function as a lysine acetyltransferase and to modify glycerol kinase (HvGlpK), an enzyme central to glycerol metabolism. Consistent with this metabolic enzyme target, we find HvPat2 to be required for long-term survival of *H. volcanii* on glycerol, but not other carbon sources like glucose. We have shown that residues predicted to be involved in the HvPat2:acetyl-CoA:substrate-binding interface play a key role in HvPat2 activity as well as the ability of cells to grow on glycerol. To expand on this work, it would be of interest to determine the crystal structure of the HvPat2 enzyme in its apo (unbound) form as well as when bound with HvGlpK to identify the complete active site of HvPat2. Understanding the molecular mechanisms used to regulate lysine acetyltransferases like HvPat2, which consist of a single GNAT domain, is also of significant interest. Whether the HVO_2384 CBS-RHF domain protein partner of HvPat2 Y154A, the UspA domain protein encoded by *hvo_1820* that overlaps the coding sequence of *pat2*, acetylation of HvPat2 K125, Sir2-mediated deacetylation, or other mechanisms are associated with this regulation remains to be determined.

## Data Availability

The MS-based proteomic data sets generated in this study were deposited in the UCSD MassIVE repository (Mass Spectrometry Interactive Virtual Environment, https://massive.ucsd.edu) under the accession number MSV000098776. Each data set includes the raw MS files used for peptide identification and quantification of the co-purifying protein partner of Pat2 Y154A compared to the empty vector control. The data sets are publicly available through the PRoteomics IDEntifications (PRIDE) database (65) under the accession number PXD067160.

## SUPPLEMENTAL MATERIAL

Figure S1 mbio.02514-25-s0001.tif Growth characteristics.

Figure S2 mbio.02514-25-s0002.tif HvGlpKEc at the Ni^2+^ purification stage.

Table S1 mbio.02514-25-s0003.pdf List of strains, plasmids, and primers used in this study.

ASM does not own the copyrights to Supplemental Material that may be linked to, or accessed through, an article. The authors have granted ASM a non-exclusive, world-wide license to publish the Supplemental Material files. Please contact the corresponding author directly for reuse.
