# Supplementary figures and images for: GNAT family Pat2 is required for long-term survival on glycerol and catalyzes lysine acetylation of glycerol kinase in hypersaline-adapted archaea
Source: mBio. 2025 Oct 27;16(11):e02514-25. doi: 10.1128/mbio.02514-25 (PMC12607858; doi:10.1128/mbio.02514-25)

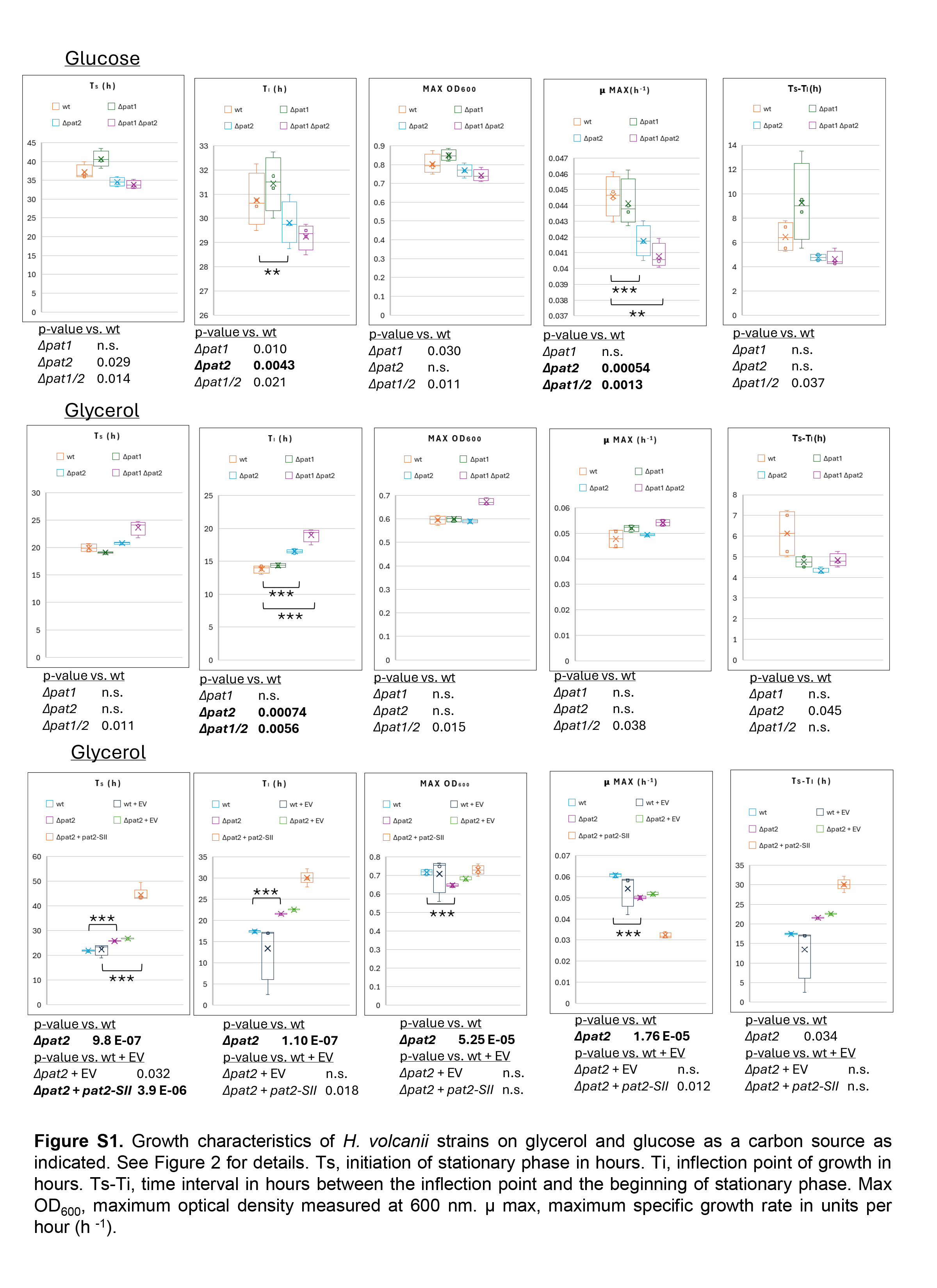

Supplement: Figure S1 — Growth characteristics. [file mbio.02514-25-s0001.tif]

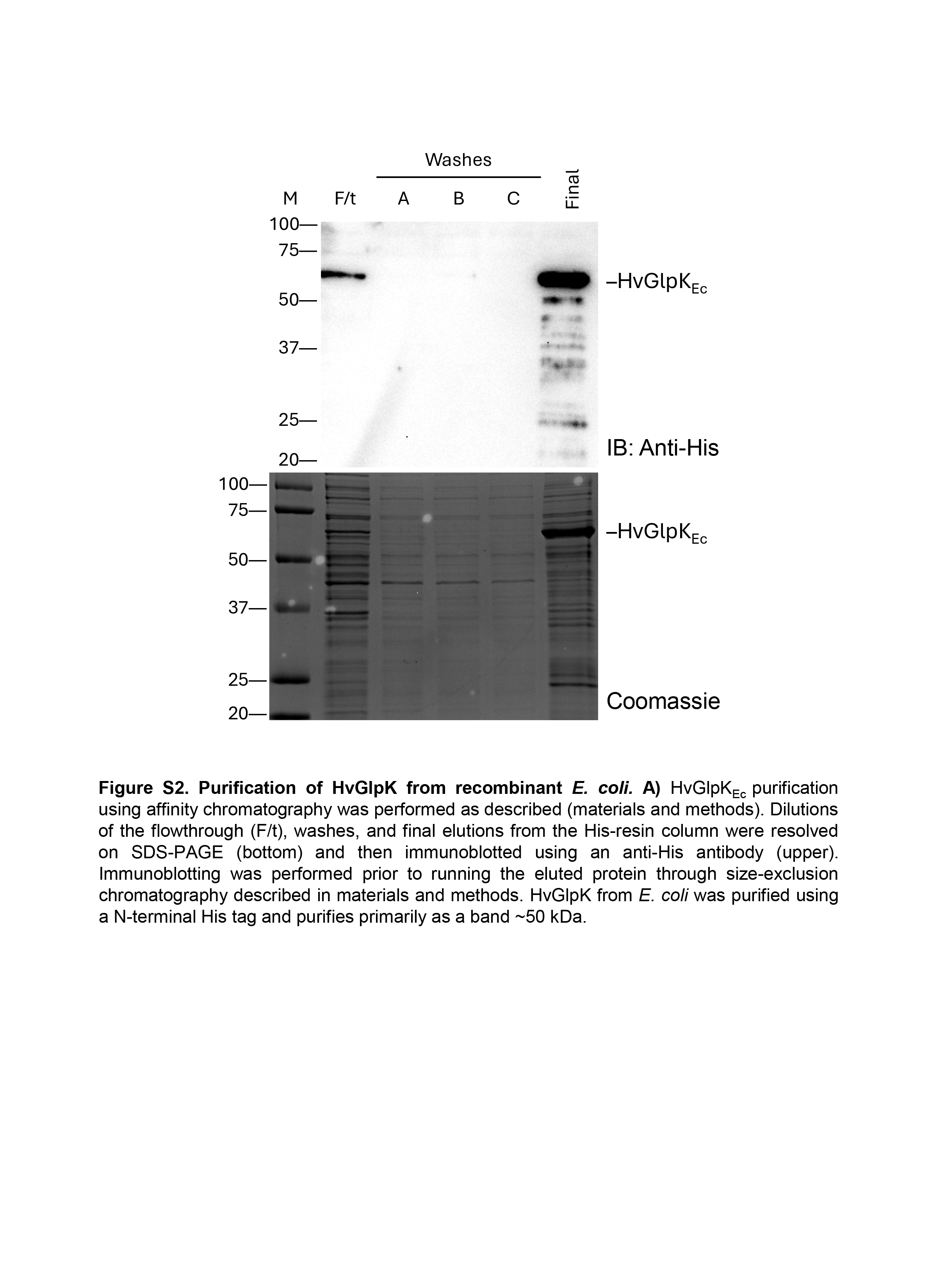

Supplement: Figure S2 — HvGlpKEc at the Ni2+ purification stage. [file mbio.02514-25-s0002.tif]
