# Supplementary material for: GNAT family Pat2 is required for long-term survival on glycerol and catalyzes lysine acetylation of glycerol kinase in hypersaline-adapted archaea
Source: mBio. 2025 Oct 27;16(11):e02514-25. doi: 10.1128/mbio.02514-25 (PMC12607858; doi:10.1128/mbio.02514-25)
Supplement: Table S1 — List of strains, plasmids, and primers used in this study. [file mbio.02514-25-s0003.pdf]

Table S1. List of strains, plasmids, and primers used in this study.

Strains	Genotype	Ref.
H. volcanii		
H26	DS70 Δ*pyrE2*	(1)
JM501	H26 Δ*pat1*	(2, 3)
JM502	H26 Δ*pat2*	(2, 3)
JM504	H26 Δ*pat1* Δ*pat2*	(2, 3)
E. coli		
Top10	F^- *mcrA* Δ(*mrr-hsdRMS-mcrBC*) Φ80*lacZ*ΔM15 Δ*lacX74 recA1 araD139* Δ*(ara-leu)*7697 *galU galK* λ^- *rpsL*(Str^R) *endA1 nupG*	Invitrogen
GM2163	F^- *ara-14 leuB6 fhuA31 lacY1 tsx78 glnV44 galK2 galT22 mcrA dcm-6 hisG4 rfbD1 rpsL136 dam13::Tn9 xylA5 mtl-1 thi-1 mcrB1 hsdR2*	New England Biolabs
BL21-CodonPlus(DE3)-RIPL (Rosetta)	B F^- *ompT hsdS*(rB^- mB^-) *dcm*$^+$ Tet^r *gal* λ(DE3) *endA* Hte [*argU proL* Cam^r] [*argU ileY leuW* $Strep/Spec^R$]	Agilent

Plasmids	Description	Ref
pTA963	Overexpression vector with 6xHis tag, *pyrE2* and *hdrB* markers, and pHV2 origin, derived from pTA962 by insertion of a His tag (CAC)6 tract	(4)
pJAM202C	Amp^R; Nv^R; pJAM202-derived control plasmid	(5)
pJAM4016	Amp^R; Nv^R; pBAP5010 carrying $P2_{rrn}$-*pat1-SII*	(5)
pJAM4017	Amp^R; Nv^R; pJAM809 containing $P2_{rrn}$-*pat2-SII*	This study
pJAM4554	pTA963-derived plasmid containing P_{tna} -*pat2-SII*	This study
pJAM4555	Amp^R; Nv^R; pJAM4017-derived plasmid encoding HvPat2(E105A)-SII	This study
pJAM4556	Amp^R; Nv^R; pJAM4017-derived plasmid encoding HvPat2(V110A)-SII	This study
pJAM4557	Amp^R; Nv^R; pJAM4017-derived plasmid encoding HvPat2(N147A)-SII	This study
pJAM4558	Amp^R; Nv^R; pJAM4017-derived plasmid encoding HvPat2(Y154A)-SII	This study
pET15B	Amp^R; pBR322-derived plasmid containing P_{T7} -6xHis tag	Novagen
pJAM4360	Amp^R; pET15B-derived plasmid containing P_{T7} -6xHis-*glpK*	This study

Primers	5'-3'	Ref.
F-glpK	GGTCATATGTCAGGAGAAACTTACGTCGG	This study
R-glpK	TTTGCTCAGCTTATTCCTCCCGTGCCC	This study
HVO_1821-NdeI	GGGACATATGAGCGACCGAACGTTCTCCGACGCC	This study
HVO_1821-KpnI	TAGGTACCGCCGCCCTCGTCGTCCG	This study
Pat2_ecori_fwd	GCCGAATTCGCTTCTAGAGCGGCCGC	This study
Pat2_noti_rev	GCGCGGCCGCGCTTAATGCGC	This study
HVPRRNF*	CGATGCCCTTAAGTACAACAGGGT	

T7TerR*	AACCCCTCAAGACCCGTTTAGAG	
Pat2_SDM-anchor1	CGTTGTATTCGGGTATCTCGTAATCTCGTGGTATCTCTCG	This study
Pat2_SDM-anchor2	GAGCGACCGAACGTTCTCCGACGCCGTCGCCGAC	This study
E105A_rev	GAAGATGGCGAGGGCGTAGGCGTCGC	This study
V110A_rev	GTACGTCTGGTGGGCGAAGATGGCGAG	This study
N147A_rev	CGCCGCGCGGGCCCAGCGCTCG	This study
Y154A_rev	CGACGGTCTGGGCGAGGCCGACCGC	This study

Abbreviations: Amp^R, ampicillin resistance; Nv^R, novobiocin resistance; -SII, C-terminal StrepII tag; *pat1* or HvPat1, HVO_1756; *pat2* or HvPat2, HVO_1821; *glpK* or HvGlpK, HVO_1541; 6xHis-, N-terminal polyhistidine tag.

Supplemental References

1. Allers T, Ngo HP, Mevarech M, Lloyd RG. 2004. Development of additional selectable markers for the halophilic archaeon *Haloferax volcanii* based on the *leuB* and *trpA* genes. Appl Environ Microbiol 70:943-53.
2. Couto-Rodríguez RL, Koh J, Chen S, Maupin-Furlow JA. 2023. Insights into the lysine acetylome of the haloarchaeon *Haloferax volcanii* during oxidative stress by quantitative SILAC-based proteomics. Antioxidants (Basel) 12:1203.
3. Weber KR, Novillo B, Maupin-Furlow JA. 2025. Revisiting synthetic lethality of Gcn5-related N-acetyltransferase (GNAT) family mutations in *Haloferax volcanii*. Microbiol Spectr 13:e0122925.
4. Allers T, Barak S, Liddell S, Wardell K, Mevarech M. 2010. Improved strains and plasmid vectors for conditional overexpression of His-tagged proteins in *Haloferax volcanii*. Appl Environ Microbiol 76:1759-69.
5. Zhou G, Kowalczyk D, Humbard M, Rohatgi S, Maupin-Furlow J. 2008. Proteasomal components required for cell growth and stress responses in the haloarchaeon *Haloferax volcanii*. J Bacteriol 190:8096-8105.
